# Dopamine receptor 1 expressing B cells exert a proinflammatory role in female patients with rheumatoid arthritis

**DOI:** 10.1038/s41598-022-09891-6

**Published:** 2022-04-08

**Authors:** Karolin Wieber, Leonie Fleige, Styliani Tsiami, Jörg Reinders, Jürgen Braun, Xenofon Baraliakos, Silvia Capellino

**Affiliations:** 1 Department of Immunology, Research Group of Neuroimmunology, IfADo-Leibniz Research Centre for Working Environment and Human Factors, Ardeystr 67, 44139 Dortmund, Germany; 2 Rheumazentrum Ruhrgebiet Herne, Ruhr University Bochum, Claudiusstr. 45, 44649 Herne, Germany; 3 Department of Toxicology, Analytical Chemistry, IfADo-Leibniz Research Centre for Working Environment and Human Factors, Ardeystr 69, 44139 Dortmund, Germany

**Keywords:** Immunology, Rheumatology

## Abstract

Rheumatoid arthritis (RA) is a chronic rheumatic disease with a clear sex-bias. Recent data indicated a role for dopamine in RA pathogenesis, while dopaminergic pathways can be modulated by estrogens. As defined mechanism of action of dopamine on B cell function in RA are unclear, we aimed to elucidate this, with special focus on sex-differences. Healthy controls (HC, n = 64) and RA patients (n = 61) were recruited. Expression of D_1_–D_5_ dopamine receptors (DRs) was investigated by flow cytometry on peripheral blood mononuclear cells (PBMCs). D_1_-like DRs were stimulated in vitro to assess effects on B cell activation and proliferation. Secretion of cytokines and dopamine content were measured by ELISA. All DRs were expressed on PBMCs of HC and RA patients. Dopamine content in PBMCs, and frequency of D_1_DR expressing B cells were significantly higher in RA females (p < 0.001). Expression of D_1_DR on RA B cells correlated positively with disease duration and severity only in women. Combined B cell and D_1_-like DR stimulation induced higher IL-8 and CCL-3 secretion from PBMCs of female RA patients compared to HC. These results indicate sex-specific differences in dopaminergic pathway in RA, with a proinflammatory feature of the D_1_DR pathway in women.

## Introduction

Rheumatoid arthritis (RA) is an autoimmune disease characterized by chronic joint inflammation, articular bone erosion and joint destruction with decrease of function in many patients^1^.

In most epidemiological studies RA affects up to 1% of the population^2,3^ and shows a clear female predominance^4^. Not only the prevalence but also the course of the disease differs between men and women. One possible explanation is the differential role of sex hormones on immune reactions^5^ but other factors such as genetic predisposition, epigenetics and gut microbiota likely also play a role^6–8^. Although the pathogenesis of RA is not entirely clear, there is no doubt that B cells play a crucial role, since anti B cell therapy with rituximab is beneficial for some patients (11) and autoantibodies such as rheumatoid factor (RF) and anti-citrullinated protein antibody (ACPA) are produced by differentiated B cells, the plasma cells^9,10^. Besides autoantibody production, B cells also present antigens to T cells and produce proinflammatory cytokines thereby worsening RA. Disease improvement after B cell depletion therapy in both seronegative and seropositive RA patients^11^ as well as other clinical evidences^12^ underline the importance of these antibody-independent B cell functions in RA^13^. For example, secretion of cytokines by activated B cells is involved in bone resorption. In this context, CCL3 which inhibits the bone matrix-producing osteoblasts^14^, and TNF-α which stimulates differentiation of osteoclasts^15^ have been identified as relevant cytokines among others.

The immune system can be affected by the nervous system and neurotransmitters. Dopamine is known as a neurotransmitter of the sympathetic nervous system that plays several important roles not only in the brain but also in the whole body. Recent evidence, however, supports a key role of dopaminergic pathways also in the modulation of immunity in the periphery^16–19^. Dopamine acts via five different dopamine receptors (DRs), which are expressed in most immune cells. In addition, immune cells are able to synthesize and utilize dopamine as an autocrine/paracrine transmitter^20–22^. DRs belong to G protein coupled receptors (GPCRs)^23,24^. Depending on their pharmacological, biochemical, and physiological profile DRs are assigned in two subclasses, namely D_1_- and D_2_-like receptors. D_1_ and D_5_ DR belong to the stimulatory D_1_-like and D_2_, D_3_ and D_4_ DR to the inhibitory D_2_-like receptor family^25^.

There are clinical evidences suggesting the involvement of the neurotransmitter dopamine in the pathogenesis of RA: the incidence of RA in Schizophrenia patients is much lower than in the general population^26^, and the prevalence of RA is also altered in Parkinson’s disease, even though the causal interaction of these diseases is conflicting^27–29^. Also, the incidence of restless legs syndrome is increased in RA patients compared to healthy controls (HC)^30^ and, of interest, patients with restless leg syndrome have increased plasma levels of dopamine^31^. Furthermore, high local concentration of dopamine was measured in the synovial fluid of RA patients^32^.

Also in vitro, dopamine modulates immune response. Previous experimental studies have suggested a pro-inflammatory role for D_1_-like DR and a rather anti-inflammatory role for D_2_-like DR, whereas the mechanisms of action have been poorly described to date. Notably, dopamine was shown to promote the interaction between B cells and T-follicular helper (T_FH_) cells in germinal centers of the lymph nodes, hereby promoting B cell maturation via D_1_DR^33^. Further, it was demonstrated that D_2_DR expression on B cells negatively correlates with disease activity as well as with inflammatory biomarkers in human RA^34^, and the migration of synovial fibroblasts from young RA patients was increased by D_1_-like DR activation in vitro^35^. Also, experimental studies in vivo strongly suggested a role for dopamine in arthritis^36–39^. However, more research is needed to better understand the role of specific DR signaling in RA.

The primary aim of this study was to investigate the possible involvement of the neurotransmitter dopamine in the systemic immune cell activation in RA^40^.

Since women have a higher incidence of RA than men and estrogens appear to have a direct influence on DR expression^41^, we investigated the expression and function of the dopaminergic pathway in peripheral immune cells of RA patients with special focus on sex differences.

## Results

### Study population

We recruited a total of 61 patients with confirmed diagnosis of RA, 64 healthy controls and 43 patients with chronic inflammatory rheumatic and musculoskeletal diseases (CIRMD): 20 psoriatic arthritis (PsA) and 23 axial spondyloarthritis (axSpA).

The mean age was 44.4 years (y) for HC and 53 years for RA, with an age range of 19–75 and 21–79 respectively. Mean age for PsA was 44.3 years, with an age range of 25–71, whereas mean age for axSpA was 44.6 years with an age range of 26–81. The mean disease duration of RA patients was 5.7 years, for PsA 5.3 years and for axSpA 12 years. Detailed subject characteristics are listed in Table 1. Patients with psychiatric or neurological comorbidities were excluded from the study.

**Table 1:** Subject characteristics.

	HC (n = 64)	RA (n = 61)	PsA (n = 20)	axSpA (n = 23)
Female (%)	62.5	64	50	43.5
Age mean (years)	44.4	53	44.3	44,6
Age range (years)	19–75	21–79	25–71	26–81
Disease duration (mean, years)	N.A	5.7	5.3	12
**Medication (% of total patients)**				
Glucocorticoids	N.A	51	45	N.A
csDMARDs	N.A	64	70	N.A
bDMARDs	N.A	49	60	91
NSAIDs	N.A	N.A	35	48

*HC* healthy controls, *RA* rheumatoid arthritis, *PsA* psoriatic arthritis, *axSpA* axial spondyloarthritis, *csDMARD* conventional synthetic disease-modifying anti-rheumatic drug, *bDMARDs* biologic disease-modifying anti-rheumatic drug, *NSAIDs* nonsteroidal antiinflammatory drugs.

### Dopaminergic pathway is expressed in PBMCs of healthy controls and rheumatoid arthritis patients

**Figure 1:**
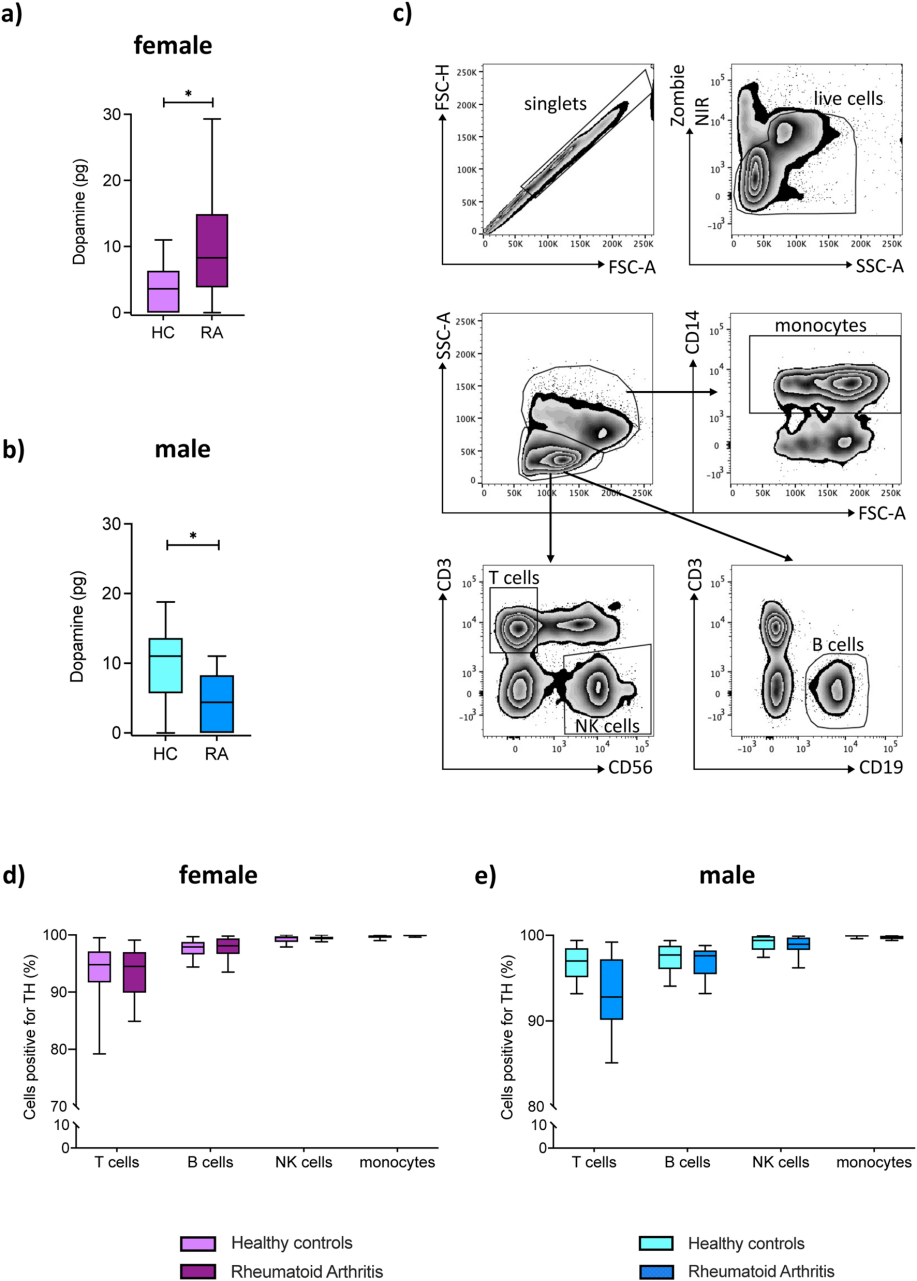
Dopamine content and expression of tyrosine hydroxylase in peripheral immune cells from RA patients and HC. Dopamine level was measured in PBMCs obtained from healthy control (HC) and rheumatoid arthritis (RA) patients by TriCat ELISA and TH expression was analyzed by flow cytometry. (**a**, **b**) Concentration of dopamine in 10^6^ freshly isolated PBMCs (HC female n = 10, RA female n = 14, HC male n = 11, RA male n = 10). (**c**) Gating strategy to discriminate between CD3^+^CD56^-^ T cells, CD3^-^CD56^+^ NK cells, CD19^+^ B cells and CD14^+^ monocytes d) Quantification of TH expression in aforementioned PBMC subsets from HC and RA patients (HC female n = 24, RA female n = 27, HC male n = 16, RA male n = 15). Welch’s t test was used to compare catecholamine levels in PBMCs from HC and RA patients; Mixed-effects analysis with Geisser-Greenhouse correction and Sidak multiple comparison test was used for comparing TH expression between groups; *p ≤ 0.05.

**Figure 2:**
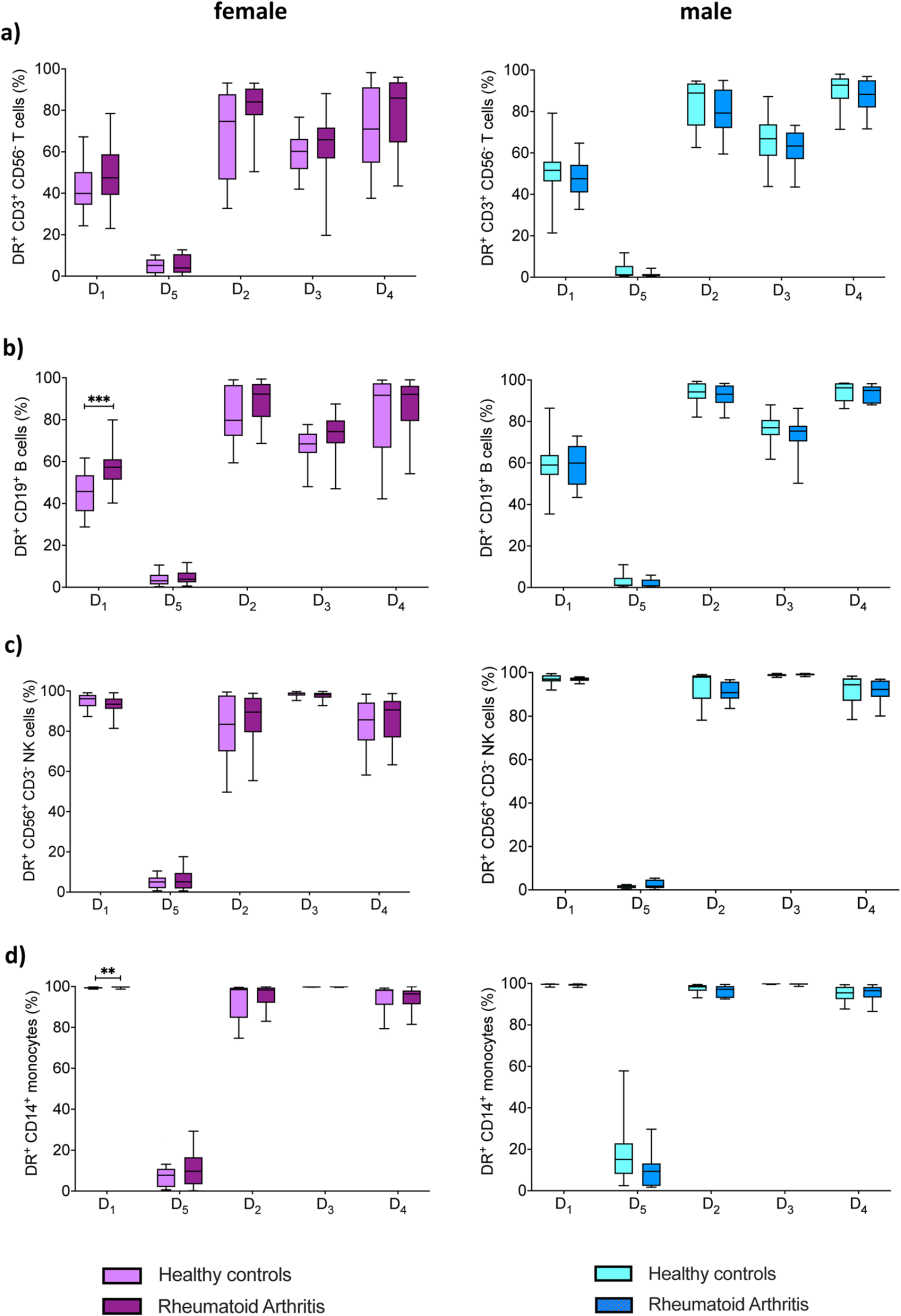
Expression of dopaminergic receptors in peripheral immune cells from RA patients and HC. Quantification of D_1_–D_5_ DR expression in CD3^+^CD56^-^ T cells (**a**), CD19^+^ B cells (**b**), CD3^-^CD56^+^ NK cells (**c**) and CD14^+^ monocytes (**d**) from HC (female n = 24, male n = 16) and RA patients (female n = 27, male n = 15). Mixed-effects analysis with Geisser-Greenhouse correction and Sidak multiple comparison test was used for comparing DR expression between groups; **p ≤ 0.01, ***p ≤ 0.001.

PBMCs from HC and RA subjects contained dopamine (Fig. 1a,b). Notably, PBMCs of female RA contained about three times more dopamine than HC (Fig. 1a), whereas PBMCs from male RA contained significant less dopamine than male HC (Fig. 1b). As dopamine is a precursor of noradrenaline and adrenaline, we measured the amount of these catecholamines as well, but found comparable concentrations in both groups (Suppl. Fig. 1), thus suggesting that only dopamine is specifically altered during RA. To find out if PBMCs synthesize dopamine themselves, we stained T cells, B cells, NK cells and monocytes for tyrosine hydroxylase (TH), the rate limiting enzyme in catecholamine synthesis. The gating strategy for these leukocyte subpopulations is shown in Fig. 1c. Our results revealed the presence of TH in all leukocytes (Fig. 1d,e), with no statistical differences in RA compared to HC. We further analyzed the expression of dopaminergic receptors (DRs) on leukocyte subpopulations. The gating strategy is shown in Supplementary Fig. 2a. We demonstrated the presence of D_1_–D_5_ DRs on all leukocyte subpopulations analyzed (Fig. 2), with NK cells and monocytes showing the highest amount of all DR^+^ cells. D_5_DR was very weakly expressed in all analyzed PBMC subsets. In most of them, D_2_-like DRs were higher expressed than D_1_-like DRs. In general, a tendency towards a higher expression of almost all DRs in all PBMC subsets was observed for RA women in comparison to HC. In contrast, rather reduced DR expression levels for RA men compared to healthy males indicated a possible sex bias. Of interest, a significant higher expression of D_1_DR was detected in B cells from female RA patients compared to HC, whereas no differences were observed in men (Fig. 2b). The gating strategy is shown in Supplementary Fig. 2a.

No differences in the D_1_DR expression on B cells compared to the HC were observed in axSpA and PsA patients (Suppl. Figs. 4 and 5). However, D_1_DR on B cells tended to be higher in diseased women compared to HC and not in men, and expression of D_5_DR was significantly lower in diseased women compared to HC for T cell, B cells and NK cells (Suppl. Figs. 4 and 5), whereas D_4_DR expression was significantly lower in T cells of axSpA male patients compared to HC and no differences were observed in women (Suppl. Fig. 4b). Based on these results, a sex-biased effect of dopamine is plausible not only in RA but also in other chronic rheumatic diseases.

### D_1_DR expression on B cells correlates with clinical parameters in RA patients

**Figure 3:**
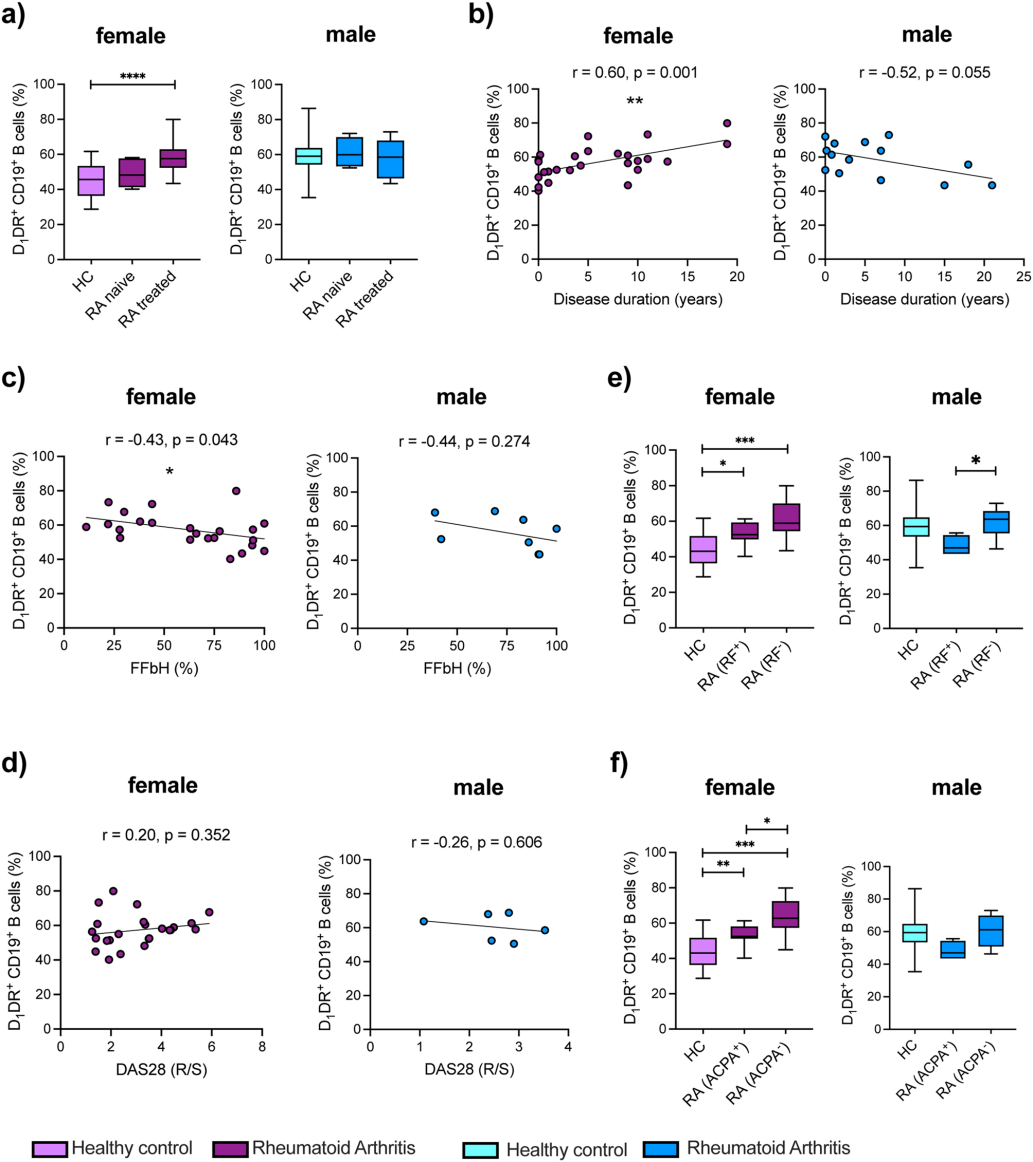
D_1_DR expression on peripheral B cells from female RA patients correlates with clinical parameters. Differential expression of D_1_DR on CD19^+^ B cells from RA patients was correlated with available patient information at day of blood withdrawal. (**a**) comparison of D_1_DR level on CD19^+^ B cells from HC (female n = 24, male n = 16) as well as naïve RA (female n = 5, male n = 4) and treated RA (female n = 20, male n = 11) patients (**b**–**d**) D_1_DR level on CD19^+^ B cells from RA patients was analyzed regarding disease duration (**b**, female n = 27, male n = 14), FFbH (**c**, female n = 27, male n = 7) and DAS28 score (**d**, female n = 27, male n = 6). (**e**, **f**) Quantification of D_1_DR expression on CD19^+^ B cells from HC (female n = 23, male n = 14) and RA patients categorized into rheumatoid factor (RF) positive and negative (**e**, female n = 14 and 13, male n = 4 and 9 respectively)) or anti-citrullinated protein antibodies (ACPA) positive and negative (**f**, female n = 16 and 10, male n = 4 and 6 respectively). Simple linear regression with Pearson correlation analysis was used to analyze D_1_DR expression in relation to clinical parameters; Brown-Forsythe and Welch ANOVA tests with Dunnett T3 multiple comparison test were used to compare D_1_DR expression between groups; *p ≤ 0.05, **p ≤ 0.01, ***p ≤ 0.001, ****p ≤ 0.0001.

To investigate a possible clinical relevance of D_1_DR on B cells in RA, we correlated its expression with clinical parameters (Fig. 3 and Suppl. Fig. 5). D_1_DR expression on B cells tended to be increased also in female naïve RA patients compared to HC, but the amount of D_1_DR + B cells is significantly higher in treated RA female patients compared to HC (Fig. 3a). No differences were observed in men (Fig. 3a). D_1_DR expression on B cells positively correlated with disease duration in female RA patients and tended to negatively correlate with disease duration in men (Fig. 3b). A negative correlation was observed between D_1_DR expression on B cells and the Hannover functional questionnaire (FFbH) (Fig. 3c), a self-assessment questionnaire encompassing functional physical limitations^42^, thus displaying a positive correlation between disease disability and D_1_DR expression. This suggests an involvement of D_1_DR-positive B cells on disease perpetuation in women. In male, no significant correlation was observed, but the number of samples available was limited (Fig. 3c). No significant correlation was found regarding disease activity score (DAS) 28 (Fig. 3d). To find out if the correlation of D_1_DR expression with disease duration is due to a physiological increase of D_1_DR on B cells during ageing, we correlated the expression of D_1_DR on B cells with age, and found no significant correlation in RA. However, a correlation was found for female HC (Suppl. Fig. 5a), therefore we decided to perform a multiple linear regression for RA patients, which includes both age and disease duration. A statistical significance between D_1_DR expression and disease duration was obtained for women (**p = 0.0044, β = 0.9339; age: p = 0.5542, β = 0.062) as well as for men (*p = 0.020, β = − 0.9767; age: p = 0.1204, β = − 0.2931). To examine present correlations of age, sex, and disease with D_1_DR expression, multivariable analyses were performed with pooled data including all diseases and both sexes. Therefore, multiple linear regression with age, sex and disease as independent variables and D_1_DR expression as the dependent variable was used. It was found that age has no impact (p = 0.2149), whereas sex (***p = 0.0001) and RA (*p = 0.0024) have a statistically significant effect on D_1_DR expression. This statistical significance was not confirmed for PsA (p = 0.1803) and SpA (p = 0.0744), however the trend towards being an influencing variable is observable as well. This strengthens the hypothesis of a direct involvement of D_1_DR expression in RA pathophysiology in women.

Of interest, D_1_DR expression was higher on B cells both from seronegative as well as from seropositive female RA patients (Fig. 3e,f), and seropositive male RA patients tended to have lower D_1_DR expression on B cells compared to HC and seronegative RA patients (Fig. 3e,f), thus indicating that D_1_DR activation on B cells might influence non-canonical proinflammatory functions of B cells such as cytokine production^13^ in female patients rather than antibody production.

### D_1_DR expression increases during B cell maturation

**Figure 4:**
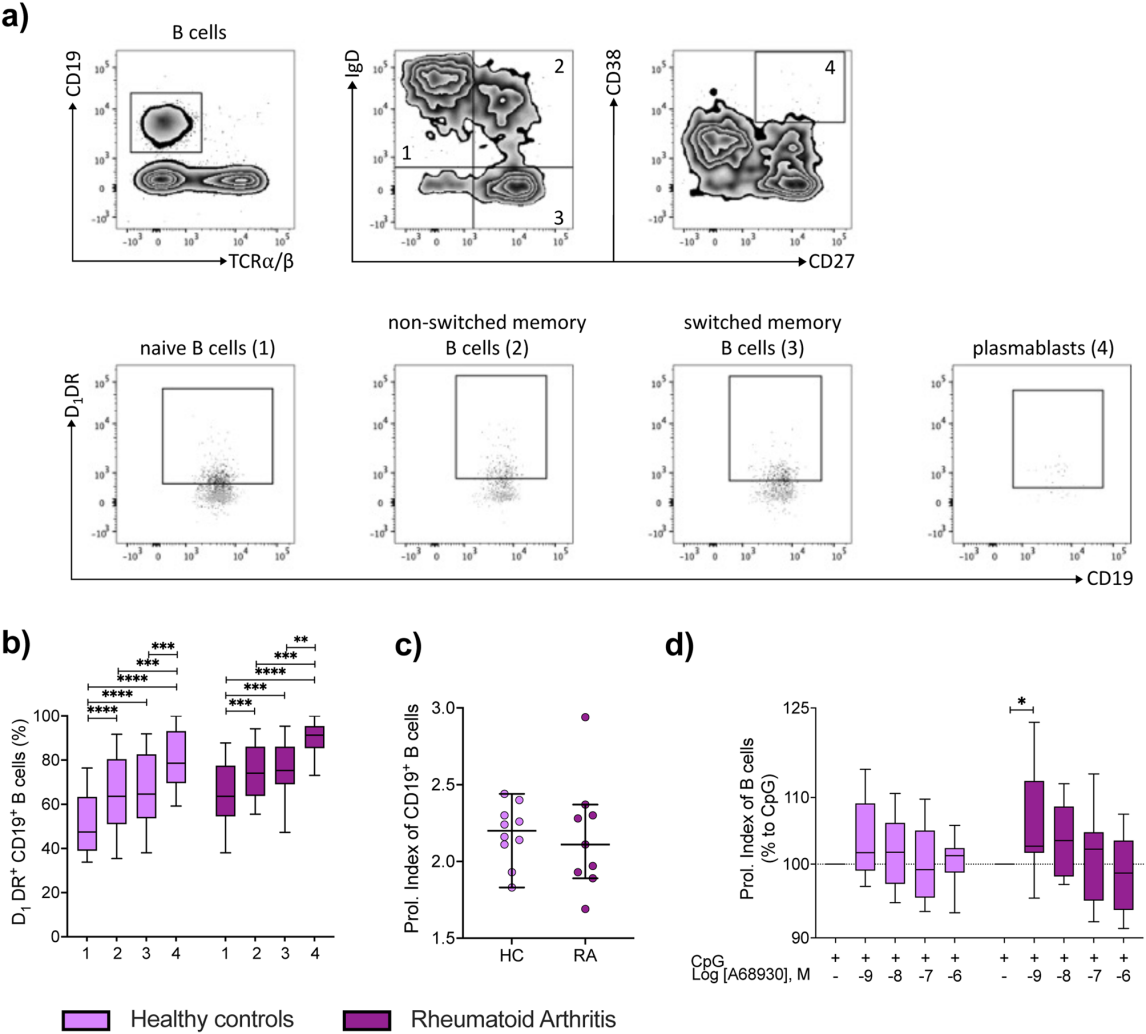
D_1_DR expression in different maturation stages of B cells and during B cell proliferation. D_1_DR expression was analyzed in defined B cell subpopulations from HC and RA patients by flow cytometry. (**a**) Gating strategy to investigate D_1_DR expression in naïve (1, CD19^+^TCRα/β^-^IgD^+^CD27^-^), non-switched memory (2, CD19^+^TCRα/β^-^IgD^+^CD27^+^), switched memory B cells (3, CD19^+^TCRα/β^-^IgD^-^CD27^+^) and plasmablasts (4, CD19^+^TCRα/β^-^CD27^+^CD38^+^), black: stained sample, grey: FMO control. (**b**) Quantification of D_1_DR on aforementioned B cell subsets from HC and RA female patients (n = 17 and 21 respectively). (**c**, **d**) PBMCs from HC and RA female patients were stimulated with CpG and indicated concentrations of D_1_-like receptor agonist A68930 for 6 days in vitro. Proliferation of CD19^+^ B cells was then analyzed by CFSE-dye dilution via flow cytometry. (**c**) Proliferation index of CD19^+^ B cells from HC and RA patients (n = 10 and 9 respectively) under pure CpG stimulation are presented as median with SD. (**d**) Proliferation index of CD19^+^ B cells of both groups after D_1_-like stimulation were normalized to CpG controls and are presented as relative changes (n = 10). One-Way ANOVA with Geisser-Greenhouse correction and Tukey multiple comparison test was used to analyze expression between B cell subsets within HC and RA group; Welch’s t test was used to compare CpG stimulated controls from HC and RA group; Raw data were logarithmized and analyzed by mixed-effects analysis with Geisser-Greenhouse correction and Dunnett multiple comparison test to determine the influence of D_1_-like receptor stimulation within the HC and RA group; *p ≤ 0.05, **p ≤ 0.01, ***p ≤ 0.001, ****p ≤ 0.0001.

As D_1_DR expression was found to be significantly increased in total CD19^+^ B cells of female RA, we next investigated D_1_DR expression in naive, non-switched memory, switched memory B cells and plasmablasts by flow cytometry. The gating strategy is shown in Fig. 4a. D_1_DR expression was higher in RA B cells already in the early maturation steps compared to HC in females (Fig. 4b). Moreover, we detected an increased D_1_DR expression during B cell maturation both in HC as well as in RA both in females and males, with plasmablasts showing the highest D_1_DR expression (Fig. 4b and Suppl. Fig. 6a). Assuming that an increase in D_1_DR has an impact on B cell function these results suggest a stronger effect on more mature B cells, not only during RA but also in the physiological conditions. Moreover, stimulation of D_1_DR in vitro with the specific agonist A68930^43^*,* in combination with CpG ODN 2006 (shortly CpG), a TLR9 ligand and thus B cell-stimulus, significantly increased B cell proliferation only in female RA patients but no significant differences were observed in HC (Fig. 4d) and in men (Suppl. Fig. 6c), even if the basal proliferation indices of the two groups were comparable (Fig. 4c and Suppl. Fig. 6b), thus indicating a stronger impact of dopamine on B cell function in female RA.

### Effects of D_1_DR stimulation on B cell regulation and activation

**Figure 5:**
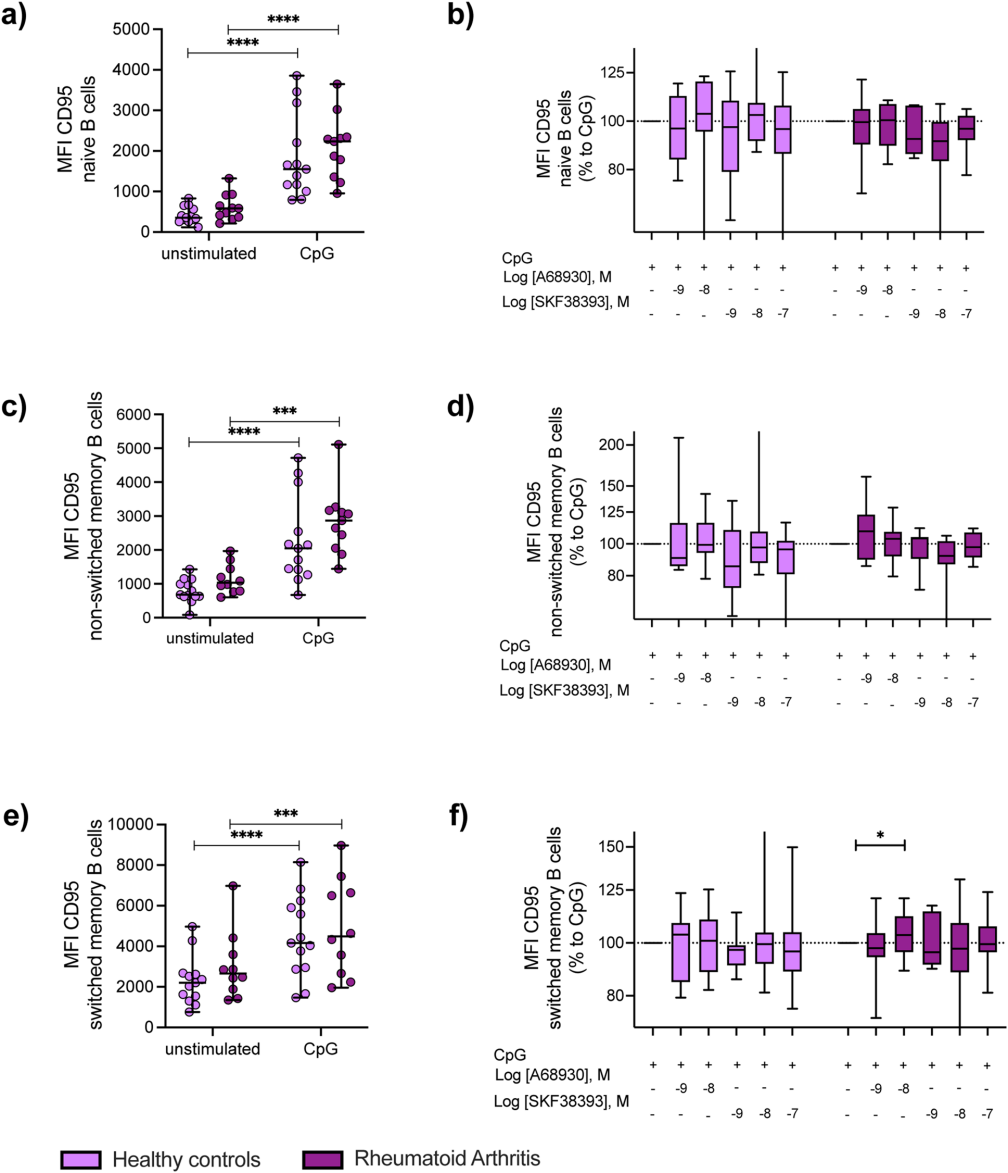
D_1_-like receptor stimulation slightly increases CD95 expression on switched memory B cells from female RA patients. PBMCs from HC (n = 13) and RA patients (n = 10–11) were stimulated with CpG and indicated concentrations of D_1_-like receptor agonists A68930 and SKF38393 for 24 h in vitro. Expression of CD95 was analyzed in naïve B cells (CD19^+^IgD^+^CD27^-^), non-switched memory B cells (CD19^+^IgD^+^CD27^+^) and switched memory B cells (CD19^+^IgD^-^CD27^+^) by flow cytometry and expression data are shown as MFI. (**a**, **c**, **e**) Absolute CD95 expression is shown for naïve B cells (**a**), non-switched memory B cells (**c**) and switched memory B cells (**e**) in unstimulated and CpG-stimulated PBMCs from HC and RA patients. Lines indicate median with SD. (**b**, **d**, **f**) CD95 expression of naïve B cells (**b**), non-switched memory B cells (**d**) and switched memory B cells (**f**) after D_1_-like stimulation was normalized to CpG controls from HC and RA patients and are presented as relative changes on a logarithmic scale. Two-Way ANOVA or mixed-effects-analysis, depending on missing values, with Sidak multiple comparison test was used to analyze CD95 expression between unstimulated and CpG stimulated samples from HC and RA; Raw data were logarithmized and analyzed by One-Way ANOVA or mixed-effects analysis, depending on missing values, with Geisser-Greenhouse correction and Dunnett multiple comparison test to determine the influence of D_1_-like receptor stimulation on CD95 expression within HC and RA group; *p ≤ 0.05, ***p ≤ 0.001, ****p ≤ 0.0001.

**Figure 6:**
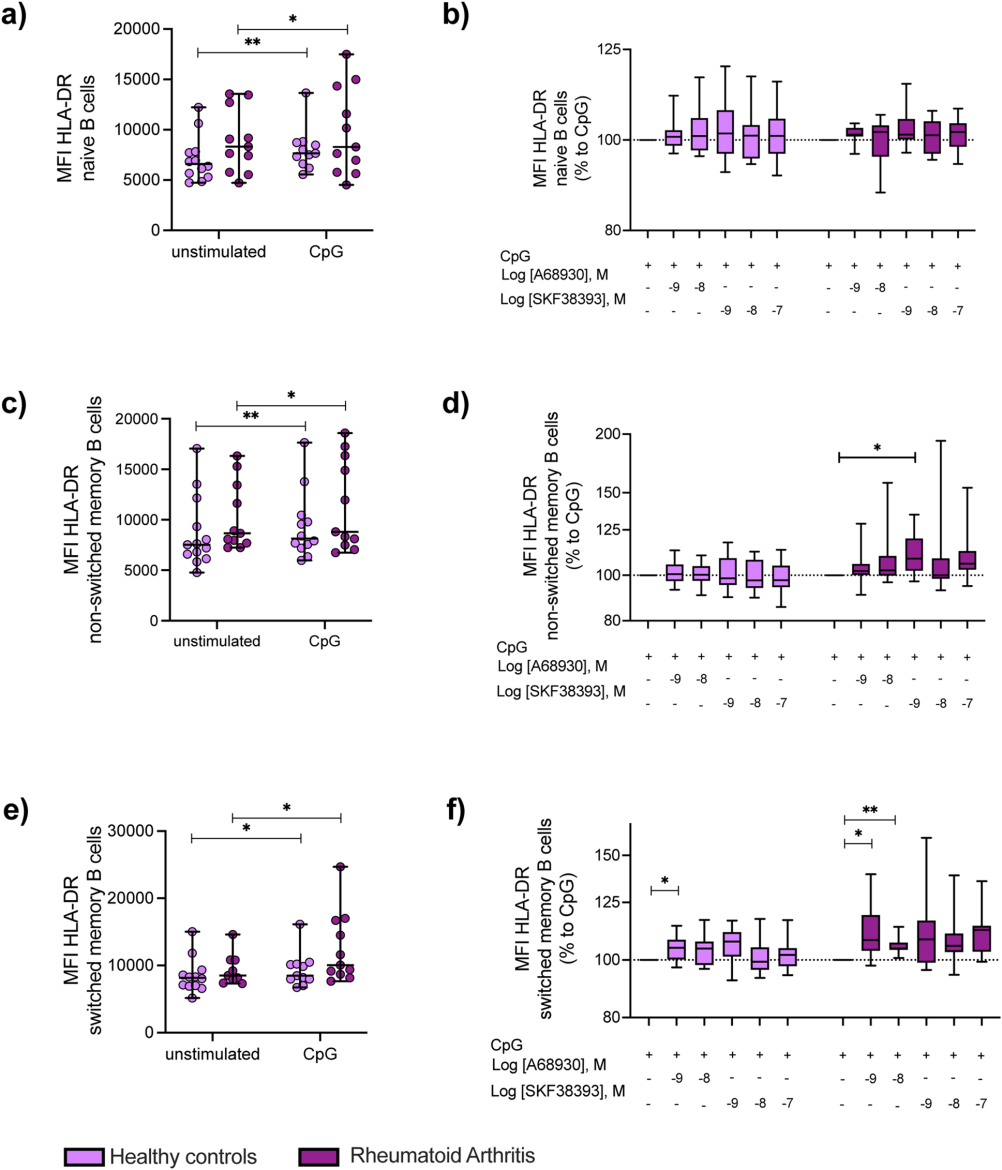
D_1_-like receptor stimulation increases activation of memory B cells from female RA patients. PBMCs from HC (n = 13–12) and RA patients (n = 11) were stimulated with CpG and indicated concentrations of D_1_-like receptor agonists A68930 and SKF38393 for 24 h in vitro. Expression of HLA-DR was analyzed in naïve B cells (CD19^+^IgD^+^CD27^-^), non-switched memory B cells (CD19^+^IgD^+^CD27^+^) and switched memory B cells (CD19^+^IgD^-^CD27^+^) by flow cytometry and expression data are shown as MFI. (**a**, **c**, **e**) Absolute HLA-DR expression is shown for naïve B cells (**a**), non-switched memory B cells (**c**) and switched memory B cells (**e**) in unstimulated and CpG-stimulated PBMCs from HC and RA patients. Lines indicate median with SD. (**b**, **d**, **f**) HLA-DR expression of naïve B cells (**b**), non-switched memory B cells (**d**) and switched memory B cells (**f**) after D_1_-like stimulation was normalized to CpG controls from HC and RA patients and are presented as relative changes on a logarithmic scale. Two-Way ANOVA or mixed-effects-analysis, depending on missing values, with Sidak multiple comparison test was used to analyze HLA-DR expression between unstimulated and CpG stimulated samples from HC and RA; Raw data of HLA-DR expression were logarithmized and analyzed by One-Way ANOVA or mixed-effects analysis, depending on missing values, with Geisser-Greenhouse correction and Dunnett multiple comparison test to determine the influence of D_1_-like receptor stimulation on HLA-DR expression within HC and RA group; *p ≤ 0.05, **p ≤ 0.01.

To further study effects of D_1_DR stimulation on B cell regulation and activation, PBMCs were stimulated in vitro with CpG, in combination or not with the D_1_-like DR agonists A68930 or SKF38393. After 24 h, expression of CD95, which regulates the death of autoreactive B cells^44^, and HLA-DR, which represents an activation marker being important for antigen presentation to T cells, were analyzed by flow cytometry in different B cell subpopulations (Figs. 5 and 6 and Suppl. Figs. 7 and 8, gating strategy in Suppl. Fig. 2b). We observed an increase of CD95 throughout maturation, with switched memory B cells showing the highest CD95 expression level being comparable between RA and HC. Also, CpG stimulation led to a significant increase of CD95 in naïve B cell, non-switched memory B cells as well as in switched memory B cells in both groups (Fig. 5a,c,e, Suppl. Fig. 7a,c,e). A significant increase in CD95 was observed only after D_1_DR stimulation in switched memory B cells from female RA (Fig. 5f), whereas expression of CD95 on naïve B cells from female RA patients tended to decrease after D_1_DR stimulation (Fig. 5b). No effects of D_1_DR stimulation were observed in men (Suppl. Fig. 7b,d,f).

Expression of HLA-DR slightly increased with B cell maturation (Fig. 6a,c,e, and Supplementary Fig. 8a,c,e). CpG treatment led to a significant higher expression of HLA-DR in all B cell subpopulations (Fig. 6a,c,e, and Supplementary Fig. 8a,c,e). D_1_DR stimulation led to significant increase of HLA-DR in non-switched memory B cells and in switched memory B cells in female RA patients and to an increase of HLA-DR expression on switched memory B cells of female HC (Fig. 6d,f). Of interest, an opposite effect was observed for switched memory B cells from male HC (Suppl. Fig. 8f) and D_1_DR stimulation did not affect HLA-DR expression in B cells from male RA patients (Suppl. Fig. 8b,d,f).

As B cells were shown to contribute to bone loss in RA by supporting osteoclastogenesis via RANKL^45^ we also aimed to investigate the effect of D_1_-like stimulation on RANKL expression. Membrane-bound RANKL as well as soluble RANKL secreted by unstimulated and of CpG-treated PBMCs were very low (data not shown), therefore no functional analyses of RANKL after D_1_DR stimulation were possible.

### D_1_DR stimulation increases IL-8 and CCL3 release in female RA

Besides RANKL secretion, B cells were also shown to promote bone loss in RA by secretion of other cytokines^14^. Thus, we further analyzed if D_1_DR stimulation has an influence on the release of proinflammatory cytokines.

**Figure 7:**
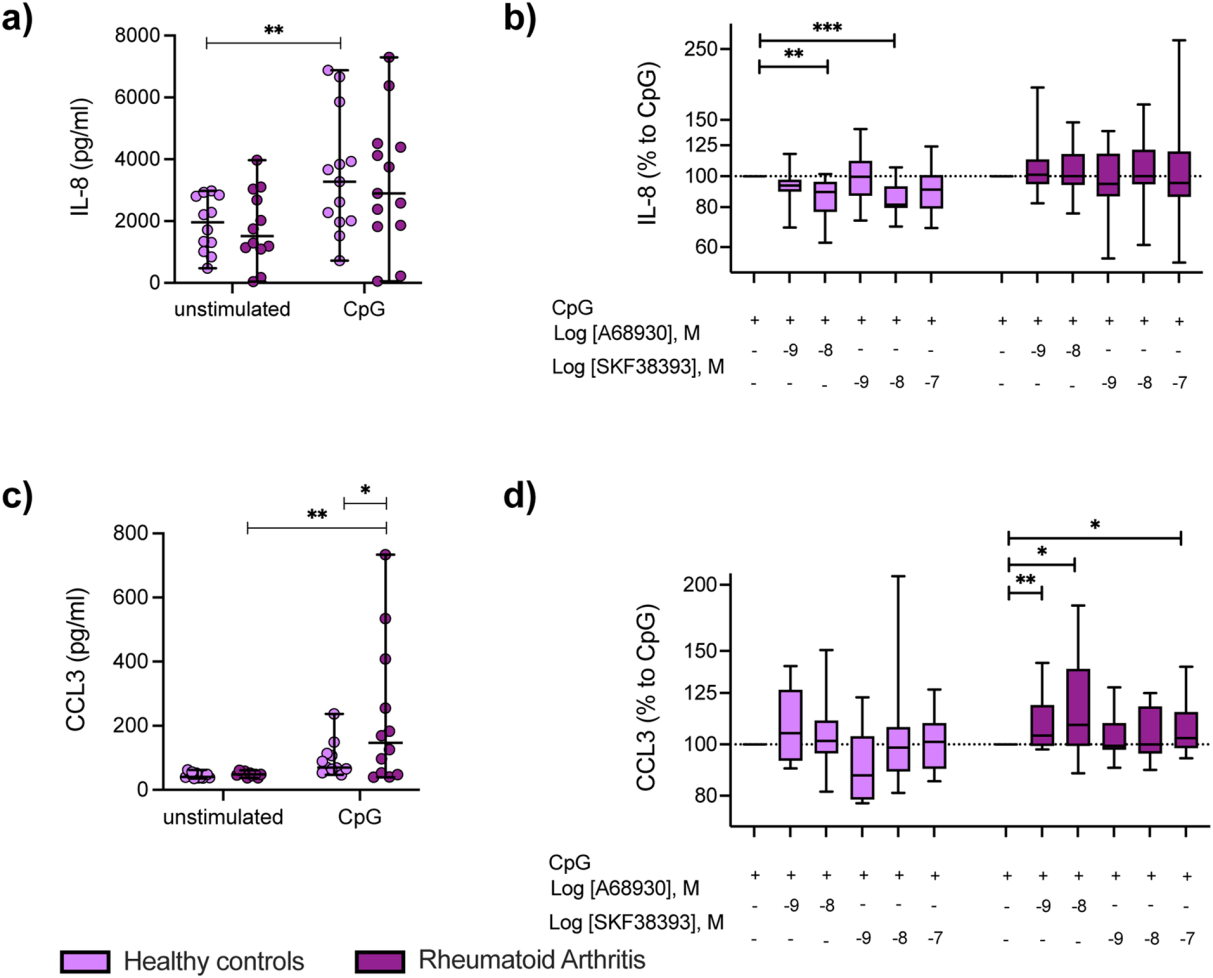
D_1_-like receptor stimulation alters cytokine secretion of PBMCs from female RA patients compared to HC. PBMCs from HC (n = 13–12) and RA patients (n = 13–12) were stimulated with CpG and indicated concentrations of D_1_-like receptor agonists A68930 and SKF38393 for 24 h in vitro. Supernatants were stored at -80 °C for subsequent analysis of IL-8 and CCL3 concentrations by ELISA. (**a**, **c**) Absolute IL-8 (**a**) and CCL3 (**c**) concentrations in supernatants from unstimulated and CpG-stimulated PBMCs from HC and RA patients are shown. Lines indicate median with SD. (**b**, **d**) IL-8 (**b**) and CCL3 (**d**) concentrations in supernatants after D_1_-like stimulation were normalized to CpG controls from HC and RA patients and are presented as relative changes on a logarithmic scale. Mixed-effects-analysis with Sidak multiple comparison test was used to analyze cytokine secretion between unstimulated and CpG stimulated samples from HC and RA; Raw data of cytokine concentrations were logarithmized and analyzed by mixed-effects analysis with Geisser-Greenhouse correction and Dunnett multiple comparison test to determine the influence of D_1_-like receptor stimulation on cytokine secretion within HC and RA group; *p ≤ 0.05, **p ≤ 0.01, ***p ≤ 0.001.

After 24 h of B cell stimulation via CpG, IL-8 release was higher in HC and tended to be higher in RA patients as well (Fig. 7a and Suppl. Fig. 9a). However, D_1_-like stimulation led to a significant decrease of IL-8 in female HC, whereas in RA patients this anti-inflammatory effect was lost (Fig. 7b). In male RA patients, an opposite, inhibitory effect of D_1_-like stimulation was observed (Suppl. Fig. 9b).

Furthermore, CCL3 release was significantly increased in CpG-treated PBMCs from RA patients but not in HC subjects (Fig. 7c and Suppl. Fig. 9c) and the D_1_-like stimulation to a significant increase of CCL3 release specifically in female RA (Fig. 7d, Suppl. Fig. 9d). Collectively, D_1_DR stimulation seems to promote cytokine secretion in B cells of RA patients.

## Discussion

Accumulating evidences suggest sex-related differences in predisposition and incidence of RA^1,4^. These findings underline the importance of a sex-specific approach to better understand RA pathophysiology, as well as for a more targeted treatment. Due to the fact that dopamine seems to be involved in RA and estrogens can modulate dopaminergic pathways, we studied here the role of dopamine in RA patients with special focus on sex differences.

Our results show that the dopaminergic receptor D_1_DR is overexpressed on B cells from female RA patients compared to HC and present its possible functional effects. The D_1_DR upregulation on RA B cells seems to be sex-specific, as we could not observe any alteration of D_1_DR expression in B cells of male RA patients compared to HC. However, a tendency to a reduced expression of all DRs in almost all PBMC subsets was found for RA men in comparison to healthy men, which underlines the overall sex bias observed in this study. Of interest, no D_1_DR alteration was observed in the other investigated rheumatic diseases PsA and axSpA, therefore we assume that a higher D_1_DR level on B cells from female RA patients is disease-specific. However, we observed some sex-specific differences in DR expression in SpA and PsA as well, therefore a sex-specific effect of dopamine on immune response is plausible also in other chronic rheumatic diseases rather than RA. Further investigations will be required. Of interest, D_1_DR expression correlated with disease duration and functional disability in RA female, thus suggesting that D_1_DR expression has a pathogenic role in disease progression and might be used as a diagnostic marker in women.

Whether the increased D_1_DR expression during RA could also be an effect of the therapy, has not been finally clarified. Indeed, treatments with DMARDs led to an improvement of the clinical features and to a higher expression of D_2_DR in RA patients in a previous study^34^. However, due to the multitude of treatment options in RA, a very large number of patients would be required in order to establish treatment-specific effects of D_1_DR expression. So far, we did not find a correlation between D_1_DR expression on B cells and current biological treatment (data not shown). We therefore assume that the increased expression of D_1_DR is probably rather related to inflammation and disease activity, as also supported by the observed positive correlation of D_1_DR expression and the disease severity parameter FFbH. The absence of a statistically significant increase in D_1_DR expression for naïve patients can probably be explained less by a lack of treatment and rather by the fact that they were newly diagnosed and have a very low disease duration. This matches the observation that D_1_DR expression increases with disease duration in treated RA women. Thus, it is unlikely that an overexpressed D_1_DR pathway is a driver in the development of RA but it might rather contribute to proinflammatory effects during the course of the disease. The reduced D_2_DR expression in RA previously described by Wei et al.^34^ could not be confirmed in our study. However, due to the opposite effects described for D_1_-like and D_2_-like receptors^24^, these results are not really in contrast with our findings. Taken together, these data support the hypothesis of an involvement of the dopaminergic pathway in the immune response in RA, suggesting that D_1_-like DRs exert a pro-inflammatory effect and D_2_-like DRs play an anti-inflammatory role at least in women. Of interest, for RA men we found a statistically significant negative correlation of D_1_DR expression with disease duration after adjustment to age and it also tended to correlate negatively with DAS28. So far, these data suggest a rather anti-inflammatory role in RA men. However, for a more expressive investigation of the impact of D_1_DR pathway in men, further studies are required.

The higher D_1_DR expression on B cells of female RA patients contributed to increased B cell proliferation. As dopamine content in inflamed RA joints is increased^37^, proliferation of pathogenic B cells could thus be accelerated locally. Higher HLA-DR expression in response to D_1_-like stimulation further suggests that dopamine may potentiate the interaction between B cells and T cells promoting inflammation. Additionally, D_1_DR activation seems to play a role for cytokine secretion after B cell activation. Our results show an increase of CCL3 after combined B cell- and D_1_-like DR stimulation of female RA PBMCs in vitro. It was previously shown that CCL3 is produced and released by B cells of RA patients and is responsible for osteoblast suppression and thus bone erosion^14^. Our results suggest therefore a possible involvement of the dopaminergic pathway on bone erosion via its activation on B cells. Also, we could observe an increase of IL-8 after D_1_DR stimulation in female RA compared to HC. This effect was due to the lack of IL-8 suppression observed in HC after B cell- and D_1_-like DR stimulation. As IL-8 could also be produced by other immune cells within the cultivated PBMCs, it is not possible so far to state if the here described change in IL-8 secretion is indirect or if B cells release IL-8. Nevertheless, due to the proinflammatory role of IL-8 in RA^46^, the effects we could observe for women seems to be of clinical relevance. In addition, rather opposite effects in cytokine production after D_1_-like stimulation were found for RA men in comparison to healthy men. Both lower IL-8 and a tendency towards lower CCL3 levels were observed for diseased men. This anti-inflammatory effect is also consistent with the sex bias we found regarding correlations of D_1_DR expression with disease parameters.

Previous studies also described TNF-α release by B cells in RA and its role on osteoblast inhibition and thus bone resorption^47^. We could not observe any significant alteration of TNF-α after D_1_DR activation (data not shown) probably due to the very strong patient-to-patient variability of TNF-α levels related to the ongoing biological treatments.

Previous studies indicated a role of dopamine in B cell maturation and immunoglobulin production^33,48^. To assess a possible effect of D_1_DR on antibody production, we performed ELISA for total IgG, but found no impact of D_1_-like receptor stimulation (data not shown). These results, together with our findings on elevated D_1_DR expression in both female seronegative and seropositive patients (Fig. 2D,E), support our hypothesis that D_1_DR might have a stronger role on cytokine-producing B cells rather than on autoantibody production in RA^13^. Nevertheless, seronegative RA patients could be positive for autoantibodies not classically included in the diagnostics, therefore a role of the dopaminergic pathway also on antibody release cannot be excluded.

This study revealed strong differences and also opposite effects of dopamine in male and female RA patients as well as in HC. These differences could be due to an effect of sex hormones on the expression of dopamine receptors, which is already suggested by other publications. For instance, Lee et al. identified a half palindromic sequence of the estrogen response element as a binding site for estrogen on the D_1_DR promoter^41^. Thus, they suggested that estrogen can act as a transcription factor on D_1_DR expression. Furthermore, in a study with ovariectomized female rats it was shown that chronic treatment with β-estradiol caused an increase in the striatal D_1_DR density^49^ supporting the hypothesis of an upregulation of D_1_DR expression by female sex hormones. However, they also described that the striatal D_1_DR density was higher in intact male rats than in female^50^, which underlines that the D_1_DR expression can be influenced differently within female and male organisms. Interestingly, this observation is in line with our findings regarding the D_1_DR level on B cells in healthy women and men. However, to the best of our knowledge nothing more is described about the connection between the hormonal and dopaminergic system in humans. Besides the production of sex hormones, also other genetic or epigenetic regulations could be involved in the observed sex bias after D_1_-like stimulation in our study. Thus, these aspects need to be further investigated.

One pitfall of our study was the cell culture of mixed PBMCs instead of isolated B cells. This was due to the very small amount of RA PBMCs (and thus B cells) available. However, due to the B cell stimulation with CpG and the short-term treatment with D_1_DR agonists, a direct involvement of B cells in the cytokine release observed is plausible.

Taken together, our results reveal a strong increase of D_1_DR expression on B cells, as well as a significant increase of dopamine in PBMCs from female RA patients, thus suggesting an involvement of the dopaminergic pathway in the immune response in these patients. The correlation between the frequency of D_1_DR + B cells and clinical parameters indicates a contribution in RA pathogenesis in women, thus suggesting the proinflammatory D_1_DR pathway as a new therapeutic target for future treatment approaches in RA women. The observed dichotomy of the dopaminergic pathway between male and female RA patients still needs to be further elucidated, but it once again underlines the importance of sex-specific studies.

## Methods

### Study cohort

The study was approved by the ethics committee of IfADo (IfADo2017/125/2019-02-04). All experiments were performed in accordance with the Declaration of Helsinki. All subjects signed informed consent for study participation.

Patients with confirmed diagnosis of RA as well as healthy controls (HC) and two cohorts of patients with other chronic inflammatory musculoskeletal rheumatic diseases (psoriasis arthritis (PsA) and axial spondyloarthritis, (axSpA, including both radiographic-SpA as well as non-radiographic-axSpA)) were recruited. Peripheral venous blood was collected in Li-Heparin tubes (Sarstedt) and processed within 6 h upon blood collection. Clinical parameters (medication, disease duration, disease activity score etc.) were provided by the clinicians after data pseudonymization.

### PBMC isolation and cell culture

Peripheral blood mononuclear cells (PBMCs) were isolated by Ficoll density gradient centrifugation (density: 1.077 g/ml; PanBiotech) and washed twice with DPBS (Gibco). Cells were resuspended in FBS (Gibco) containing 10% DMSO (Merck) and gradually frozen to -180 °C. For cell culture experiments, PBMCs were thawed, washed twice with DPBS, resuspended in B cell medium consisting of IMDM with L-Glutamine and 25 mM HEPES supplemented with 10% FBS, 1% Penicillin/Streptomycin, 1 mM Sodium Pyruvate, 1% MEM non-essential aminoacids (all from Gibco) and 0.055 mM β-Mercaptoethanol (Carl Roth) and seeded at a concentration of 0.25 × 10^6^ cells/well into 96-well round bottom plates. Cells were rested for 2 h at 37 °C and 5% CO_2_ in a humified incubation chamber, if not differently described.

To analyze the effect of D_1_-like receptor stimulation on cytokine release, 0.25 × 10^6^ PBMCs were seeded per well of a 96-well round bottom plate and stimulated with CpG ODN 2006 (0.35 μM, InvivoGen) with or without indicated concentrations of the agonists A68930 (Tocris) and SKF38393 (Tocris) for 24 h. Afterwards, cells were centrifuged and supernatants were frozen at − 80 °C until analysis.

### Flow cytometry

After thawing, PBMCs were washed twice in PBS and then stained with Zombie NIR Fixably Viability dye (BioLegend) in PBS. Unspecific binding was then blocked by incubation with 2% BSA (Carl Roth). Cells were stained extracellularly with optimally diluted antibodies in FACS buffer (2% FBS in PBS). For intracellular stainings samples were fixed with 2% Formaldehyde (Carl Roth) in FACS buffer. Afterwards cells were permeabilized and then stained intracellularly for D_2_DR, D_4_DR or TH. Unconjugated antibodies were labeled with a secondary PE-labeled donkey anti rabbit antibody. All antibodies used and the staining panels are listed in Supplementary Table 1, and a detailed description of the method is included in “Supplementary material”.

Cells were analyzed on a BD LSR Fortessa. Doublets and dead cells were already excluded at acquisition so at least 100,000 events were recorded in the live gate, whenever possible. Data were analyzed with FlowJo Version 10.3. FlowJo Version 8.87 was used for analysis of B cell proliferation.

For these multi-color panels, gates were set based on appropriate fluorescence minus one (FMO) controls, which include all antibodies of interest except one. The gating strategies are shown in Supplementary Fig. 1.

### Dopamine quantification via ELISA

For quantification of catecholamine content, 1 × 10^6^ freshly isolated PBMCs were centrifuged at 400×*g*, 4 °C for 10 min, supernatant was discarded and pellet was frozen at − 80 °C. TriCat ELISA was performed as indicated by the manufacturer (IBL). This kit allowed also to measure norepinephrine and epinephrine, which are also active catecholamines synthesized from dopamine. Briefly, cell pellets were lysed in 100 μl 0.1 M HClO_4_ with 100 μM ascorbic acid. Thereafter, catecholamines were extracted. Limits of detection were 4, 8 and 20 pg for dopamine, norepinephrine and epinephrine, respectively.

### Cell proliferation

Proliferation was analyzed by CFSE-dye dilution. PBMCs were thawed, washed once with DPBS, and stained with the Vybrant CFDA-SE Cell Tracer Dye. 1 × 10^6^ cells were resuspended in 1 ml DPBS/0.5 μM CFDA-SE and stained for 30 min at 37 °C, 5% CO_2_. Excess staining was blocked with medium containing 32.5% FBS. After centrifugation CFSE-stained cells were resuspended in pre-warmed B cell medium. 0.25 × 10^6^ cells were seeded per well of a 96-well plate and stimulated with 0.35 μM CpG ODN 2006 (InvivoGen) and indicated concentrations of D_1_-like receptor agonist A68930 (Tocris). Cells were cultured for 6 days at 37 °C, 5% CO_2_. Afterwards B cell proliferation was analyzed by flow cytometry. Protocol was adapted from Marasco et al.^51^.

### Cytokine quantification via ELISA

For quantification of secreted IL-8 and CCL3, human IL-8 ELISA MAX Standard Sets (BioLegend) and human CCL3 uncoated ELISA Kit (Invitrogen) were used, respectively. Assays were performed as described by the manufacturers. Samples were measured in duplicates or in single detection depending on sample availability. Optimal dilutions of supernatants were determined in preliminary assays.

### Statistical analysis

Statistical analysis was performed with Prism 8 software (GraphPad, v 8.3.0, www.graphpad.com). Numbers of investigated subjects are indicated in figures for each experiment. Outliers were identified and removed by ROUT method (Q = 0.01). Non-equal variability of differences regarding SDs and sphericity were computed for each comparison of two (t-test) or more groups (ANOVA or mixed-effects analysis). Pearson analysis was used for correlations. A detailed description of statistical analysis is included in “Supplementary material”. Single dots represent individual values. Box plots show mean value, 25th percentile, 50th percentile (median) 75th percentile, and maximum value.

### Ethics approval and consent to participate

The study was approved by the ethics committee of IfADo (IfADo2017/125/2019-02-04). All subjects signed informed consent for study participation.

## Supplementary Information

Supplementary Information.

## Data Availability

The datasets used and/or analyzed during the current study are available from the corresponding author on reasonable request.

## Abbreviations

RA: Rheumatoid arthritis
HC: Healthy controls
PsA: Psoriasis arthritis
axSpA: Axial spondyloarthritis
DR: Dopaminergic receptor
PBMCs: Peripheral blood mononuclear cells
IL: Interleukin
TNF: Tumor necrosis factor
CCL: C–C motif chemokine ligand
DMARD: Disease-modifying anti-rheumatic drug
RANKL: Receptor activator of nuclear factor kappa B ligand
HLA: Human leukocyte antigen
CD: Cluster of differentiation
TH: Tyrosine hydroxylase
FFbH: Hannover functional questionnaire
DAS: Disease activity score
