# Supplementary material for: Dopamine receptor 1 expressing B cells exert a proinflammatory role in female patients with rheumatoid arthritis
Source: Sci Rep. 2022 Apr 8;12:5985. doi: 10.1038/s41598-022-09891-6 (PMC8993840; doi:10.1038/s41598-022-09891-6)
Supplement: Supplementary file 1 — Supplementary Information. [file 41598_2022_9891_MOESM1_ESM.pdf]

# Dopamine receptor 1 expressing B cells exert a proinflammatory role in female patients with rheumatoid arthritis

Karolin Wieber[1#], Leonie Fleige[1#], Styliani Tsiami[2], Jörg Reinders[3], Jürgen Braun[2], Xenofon Baraliakos[2], Silvia Capellino[1*]

1- IfADo- Leibniz Research Centre for Working Environment and Human Factors, Department of Immunology, Research Group of Neuroimmunology. Dortmund, Ardeystr 69, 44139, Germany
2- Rheumazentrum Ruhrgebiet Herne, Ruhr University Bochum, Claudiusstr. 45, 44649, Germany.
3- IfADo- Leibniz Research Centre for Working Environment and Human Factors, Department of Toxicology, Analytical chemistry. Dortmund, Ardeystr 69, 44139, Germany

[#] these authors contributed equally

*corresponding author:

Silvia Capellino, PhD
Research Group of Neuroimmunology
Department of Immunology
IfADo - Leibniz Research Centre for Working Environment and Human Factors
Ardeystrasse 67
44139 Dortmund
Germany
Tel +49 231 1084 420
Email: capellino@ifado.de

**Supplementary material**

**Materials and methods**

**Flow cytometry**

0.25-0.5*$10^6$ cells were dispensed per well of a 96-well V-bottom plate and kept at 4°C. All incubation steps were performed in the dark. Cells were first stained with Zombie NIR Fixably Viability dye (BioLegend) in PBS for 20 min at 4°C. Unspecific binding was blocked by incubation with 2% BSA (Carl Roth) in PBS for 20 min at 4°C. Cells were stained extracellularly with optimally diluted antibodies in FACS buffer (2% FBS in PBS) for 20 min at 4°C. All antibodies used are listed in supplementary table 1. For intracellular stainings samples were fixed with 2% Formaldehyde (Carl Roth) in FACS buffer for 10 min at RT. Afterwards cells were permeabilized with FACS Permeabilizing Solution 2 (Becton Dickinson) for 10 min at RT. After another blocking step with 2% BSA in PBS for 20 min at RT, $D_2$ DR (LS-Bio), $D_4$ DR (Biorbyt) or TH (Bioss) were stained intracellularly for 20 min at RT. Unconjugated antibodies were labeled with a secondary PE-labeled donkey anti rabbit antibody (BioLegend) for 20 min at RT. Cells were analyzed on a BD LSR Fortessa. Doublets and dead cells were already excluded at acquisition so at least 100.000 events were recorded in the live gate, whenever possible. Data were analyzed with FlowJo Version 10.3. FlowJo Version 887 was used for analysis of B cell proliferation.

For these multi-color panels, gates were set based on appropriate fluorescence minus one (FMO) controls, which include all antibodies of interest except one. The gating strategies are shown in supplementary figure 1.

**Statistical analysis**

Statistical analysis was performed with Prism 8 software (GraphPad, v 8.3.0, www.graphpad.com). Numbers of investigated subjects are indicated in figures for each experiment. Outliers were identified and removed by ROUT method (Q = 0.01). Unpaired t test with Welch's correction was used to analyze two independent groups (RA and HC)

assuming non-equal SDs. Mixed-effects analysis with Geisser-Greenhouse correction - to adjust for lack of sphericity - and Sidak multiple comparison test was used to compare expression data of one DR between HC and RA/PsA/SpA in an experimental design investigating expression of $D_1$-$D_5$ DR. Simple linear regression with Pearson correlation analysis was chosen to analyze $D_1$/$D_3$ DR expression in relation to age or clinical parameters. For multivariable analyses multiple linear regression was performed including age, sex, disease and/or disease duration as independent variables and $D_1$ DR expression as the dependent variable.

Brown-Forsythe and Welch ANOVA tests with Dunnett T3 multiple comparison test were used to compare $D_1$ DR expression in three independent groups (HC, RA $RF^+$, RA $RF^-$). One-Way ANOVA with Geisser-Greenhouse correction and Tukey multiple comparison test was chosen for statistical comparison of $D_1$ DR expression in all four B cell subsets with each other. For statistical comparison of data normalized to a control group raw data were log-transformed and analyzed by One-Way ANOVA or mixed-effects analysis, depending on missing values, with Geisser-Greenhouse correction and Dunnett multiple comparison test always related to the control to determine the influence of $D_1$-like receptor stimulation within HC or RA group. Two-Way ANOVA or mixed-effects-analysis, depending on missing values, with Sidak multiple comparison test was used to compare unstimulated and CpG stimulated samples from HC and RA. $^{*}p \leq 0.05$, $^{**}p \leq 0.01$, $^{***}p \leq 0.001$, $^{****}p \leq 0.0001$. Single dots represent individual values. Box plots show 10th, 25th, 50th (median), 75th, and 90th percentile.

**Supplementary table 1:** List of all antibodies used for FACS staining

| Panel | Antigen | Clone | Fluorochrome | Company | Dilution 1/x |
|---|---|---|---|---|---|
| D1-5DR and TH in PBMC subtypes | Live/dead | - | Zombie NIR | Biolegend | 1000 |
| | CD3 | UCHT1 | PerCP | Biolegend | 200 |
| | CD56*<br>CD56 | NCAM16.2<br>MEM-188 | BV421<br>PE | BD<br>Biolegend | 100<br>200 |
| | CD19 | HIB19 | BV510 | Biolegend | 200 |
| | CD14 | M5E2 | BV650 | Biolegend | 400 |
| | $D_1$ DR | Polyclonal (rabbit) | FITC | Bioss | 50 |
| | $D_2$ DR | Polyclonal (rabbit) | - | LSBio | 200 |
| | $D_3$ DR | Polyclonal (rabbit) | Cy5 | Bioss | 100 |
| | $D_4$ DR | Polyclonal (rabbit) | - | Biorbit | 200 |
| | $D_5$ DR*<br>$D_5$ DR | #889022<br>#889022 | PE<br>AF405 | R&D Systems | 100 |
| | Donkey anti-rabbit | Poly4064 | PE | Biolegend | 400 |
| | TH | Polyclonal (rabbit) | AF647 | Bioss | 100 |
| D1DR in B cell subsets | Live/dead | - | Zombie NIR | Biolegend | 1000 |
| | CD19 | HIB19 | BV510 | Biolegend | 200 |
| | TCRα/β | IP26 | PE/Cy5 | Biolegend | 400 |
| | IgD | IA6-2 | PE | Biolegend | 800 |
| | CD27 | M-T271 | PE/Cy7 | Biolegend | 400 |
| | CD38 | HB-7 | BV650 | Biolegend | 400 |
| | $D_1$ DR | Polyclonal (rabbit) | FITC | Bioss | 50 |
| B cell proliferation | Live/dead | - | Zombie NIR | Biolegend | 1000 |
| | CFDA-SE | - | - | Thermo Fisher | - |
| | CD19 | HIB19 | BV421 | Biolegend | 200 |
| | CD27 | M-T271 | PE/Cy7 | Biolegend | 400 |
| | CD38 | HB-7 | BV650 | Biolegend | 400 |
| B cell activation markers | Live/dead | - | Zombie NIR | Biolegend | 1000 |
| | CD3 | UCHT1 | PerCP | Biolegend | 200 |
| | CD19 | HIB19 | BV510 | Biolegend | 200 |
| | IgD | IA6-2 | BV421 | Biolegend | 400 |
| | CD27 | M-T271 | PE/Cy7 | Biolegend | 400 |
| | CD38 | HB-7 | BV650 | Biolegend | 400 |
| | HLA-DR | L243 | PE | Biolegend | 400 |
| | CD95 | DX2 | PE/Dazzle$^{TM}$ 594 | Biolegend | 200 |
| | RANKL | MIH24 | APC | Biolegend | 200 |

** $D_5$ DR-PE antibody was discontinued during this study and was then replaced with the same clone but AF405 conjugated for n= 14 HC subjects, n=12 RA patients, n=6 PsA patients and n=3 SpA patients. The new antibody was stained in a separate panel, without further DR antibodies and with PE-labelled CD56 antibody instead of the BV421-conjugated one. All other antibodies were used as described above. The two different settings led to comparable results.*

**Figure legends of supplementary figures**

**Supplementary Figure 1: Norepinephrine and epinephrine levels in PBMCs from rheumatoid arthritis (RA) patients compared to healthy controls (HC).** Norepinephrine and epinephrine levels were measured in $10^6$ freshly isolated PBMCs from HC and RA patients by TriCat ELISA **a**) Concentration of norepinephrine and epinephrine in women (HC n = 11, RA n=14). **b**) Concentration of norepinephrine and epinephrine in men (HC n =11, RA n=10). Welch's t test was used to compare catecholamine levels in PBMCs from HC and RA patients.

**Supplementary Figure 2: Gating strategies of flow cytometry data. a**) Gating strategy to investigate frequency of $D_1$ - $D_5$ $DR^+$ expression as wells as TH expression exemplary in $CD19^+$ B cells, black: stained sample, grey: FMO control. **b**) Gating strategy to identify naïve B cells ($CD19^+IgD^+CD27^-$, light grey), non-switched memory B cells ($CD19^+IgD^+CD27^+$, grey) and switched memory B cells ($CD19^+IgD^-CD27^+$, dark grey). Histogram of CD95 expression is shown for all aforementioned B cell subsets.

**Supplementary Figure 3: DR and TH expression in PBMCs in male and female Psoriasis Arthritis (PsA) patients compared to HC.** $D_1$-$D_5$ DR and TH expression were analyzed in $CD3^+CD56^-$ T cells, $CD3^-CD56^+$ NK cells, $CD19^+$ B cells and $CD14^+$ monocytes from HC (female n=24, male n=16) and PsA patients (female n=10, male n=10) subjects by flow cytometry. **a**) TH expression in female (left) and male (right) subjects. **b-e**) $D_1$-$D_5$ DR expression in female (left) and male (right) subjects.

Subjects were age-matched. Outliers were identified and removed by ROUT method (Q = 0.01) as indicated in the sample size. Mixed-effects analysis with Geisser-Greenhouse correction and Sidak multiple comparison test was used for analysing TH and DR expression between groups; *$p \leq 0.05$, **$p \leq 0.01$, ***$p \leq 0.001$.

**Supplementary Figure 4: DR and TH expression in PBMCs in male and female Spondylitis Ankylosans (SpA) patients compared to HC.** $D_1$-$D_5$ DR and TH expression were analyzed in

$CD3^+CD56^-$ T cells, $CD3^-CD56^+$ NK cells, $CD19^+$ B cells and $CD14^+$ monocytes from HC (female n=24, male n=16) and SpA patients (female n=10, male n=13) by flow cytometry. **a**) TH expression in female (left) and male (right) subjects. **b-e)** $D_1$-$D_5$ DR expression in female (left) and male (right) subjects.

Subjects were age-matched. Outliers were identified and removed by ROUT method (Q = 0.01) as indicated in the sample size. Mixed-effects analysis with Geisser-Greenhouse correction and Sidak multiple comparison test was used for analysing TH and DR expression between groups; $*p \leq 0.05$, $**p \leq 0.01$.

**Supplementary Figure 5: $D_1$ DR expression on peripheral B cells correlates with age in healthy female subjects but not in RA women.** Differential expression of $D_1$ DR on $CD19^+$ B cells from female (a, HC n=24, RA n=26) and male (b, HC n=16, RA n=14) was correlated with age.

Outliers were identified and removed by ROUT method (Q = 0.01). Single dots represent individual values. Simple linear regression with Pearson correlation analysis was used to analyse $D_1$ DR and $D_3$ DR expression in relation to age and clinical parameters; $*p \leq 0.05$.

**Supplementary Figure 6: $D_1$ DR expression in different maturation stages of B cells and during B cell proliferation.** $D_1$ DR expression was analyzed in defined B cell subpopulations from male HC and RA patients by flow cytometry. **a**) Quantification of $D_1$ DR on naïve (1), non-switched memory (2), switched memory B cells (3) and plasmablasts (4) from HC and RA male patients (n = 10 and 9 respectively). **b-c**) PBMCs from HC and RA male patients were stimulated with CpG and indicated concentrations of $D_1$-like receptor agonist A68930 for 6 days *in vitro*. Proliferation of $CD19^+$ B cells was then analyzed by CFSE-dye dilution via flow cytometry. **b**) Proliferation index of $CD19^+$ B cells from HC and RA patients (n =5 and 7 respectively) under pure CpG stimulation are presented as median with SD. **c**) Proliferation index of $CD19^+$ B cells of both groups after $D_1$-like stimulation were normalized to CpG controls and are presented as relative changes (n =5 and 7 respectively).

One-Way ANOVA with Geisser-Greenhouse correction and Tukey multiple comparison test was used to analyze expression between B cell subsets within HC and RA group; Welch's t test was used to compare CpG stimulated controls from HC and RA group; Raw data were logarithmized and analyzed by mixed-effects analysis with Geisser-Greenhouse correction and

Dunnett multiple comparison test to determine the influence of $D_1$-like receptor stimulation within the HC and RA group; *$p \leq 0.05$, **$p \leq 0.01$.

**Supplementary Figure 7: $D_1$-like receptor stimulation does not affect CD95 expression on switched memory B cells from male RA patients.** PBMCs from HC (n=13) and RA patients (n=12) were stimulated with CpG and indicated concentrations of $D_1$-like receptor agonists A68930 and SKF38393 for 24 h *in vitro*. Expression of CD95 was analyzed in naïve B cells, non-switched memory B cells and switched memory B cells by flow cytometry and expression data are shown as MFI. **a, c, e**) Absolute CD95 expression is shown for naïve B cells (a), non-switched memory B cells (c) and switched memory B cells (e) in unstimulated and CpG-stimulated PBMCs from HC and RA patients. Lines indicate median with SD. **b, d, f**) CD95 expression of naïve B cells (b), non-switched memory B cells (d) and switched memory B cells (f) after $D_1$-like stimulation was normalized to CpG controls from HC and RA patients and are presented as relative changes on a logarithmic scale.

Two-Way ANOVA or mixed-effects-analysis, depending on missing values, with Sidak multiple comparison test was used to analyze CD95 expression between unstimulated and CpG stimulated samples from HC and RA; Raw data were logarithmized and analyzed by One-Way ANOVA or mixed-effects analysis, depending on missing values, with Geisser-Greenhouse correction and Dunnett multiple comparison test to determine the influence of $D_1$-like receptor stimulation on CD95 expression within HC and RA group; **$p \leq 0.01$, ***$p \leq 0.001$.

**Supplementary Figure 8: $D_1$-like receptor stimulation decreases slightly activation of memory B cells from healthy men.** PBMCs from HC (n=13) and RA patients (n=12) were stimulated with CpG and indicated concentrations of $D_1$-like receptor agonists A68930 and SKF38393 for 24 h *in vitro*. Expression of HLA-DR was analyzed in naïve B cells, non-switched memory B cells and switched memory B cells by flow cytometry and expression data are shown as MFI. **a, c, e**) Absolute HLA-DR expression is shown for naïve B cells (a), non-switched memory B cells (c) and switched memory B cells (e) in unstimulated and CpG-stimulated PBMCs from HC and RA patients. Lines indicate median with SD. **b, d, f**) HLA-DR expression of naïve B cells (b), non-switched memory B cells (d) and switched memory B cells (f) after $D_1$-like stimulation was normalized to CpG controls from HC and RA patients and are presented as relative changes on a logarithmic scale.

Two-Way ANOVA or mixed-effects-analysis, depending on missing values, with Sidak multiple comparison test was used to analyze HLA-DR expression between unstimulated and CpG stimulated samples from HC and RA; Raw data of HLA-DR expression were logarithmized and analyzed by One-Way ANOVA or mixed-effects analysis, depending on missing values, with Geisser-Greenhouse correction and Dunnett multiple comparison test to determine the influence of $D_1$-like receptor stimulation on HLA-DR expression within HC and RA group; **p ≤ 0.01, ***p ≤ 0.001, ****p ≤ 0.0001.

**Supplementary Figure 9: $D_1$-like receptor stimulation alters slightly cytokine secretion of PBMCs from male RA patients compared to HC.** PBMCs from HC (n=9-11) and RA patients (n=9-12) were stimulated with CpG and indicated concentrations of $D_1$-like receptor agonists A68930 and SKF38393 for 24 h *in vitro*. Supernatants were stored at -80 °C for subsequent analysis of IL-8 and CCL3 concentrations by ELISA. **a, c**) Absolute IL-8 (a) and CCL3 (c) concentrations in supernatants from unstimulated and CpG-stimulated PBMCs from HC and RA patients are shown. Lines indicate median with SD. **b, d**) IL-8 (b) and CCL3 (d) concentrations in supernatants after $D_1$-like stimulation were normalized to CpG controls from HC and RA patients and are presented as relative changes on a logarithmic scale.

Mixed-effects-analysis with Sidak multiple comparison test was used to analyze cytokine secretion between unstimulated and CpG stimulated samples from HC and RA; Raw data of cytokine concentrations were logarithmized and analyzed by mixed-effects analysis with Geisser-Greenhouse correction and Dunnett multiple comparison test to determine the influence of $D_1$-like receptor stimulation on cytokine secretion within HC and RA group; *p ≤ 0.05, **p ≤ 0.01, ****p ≤ 0.0001.

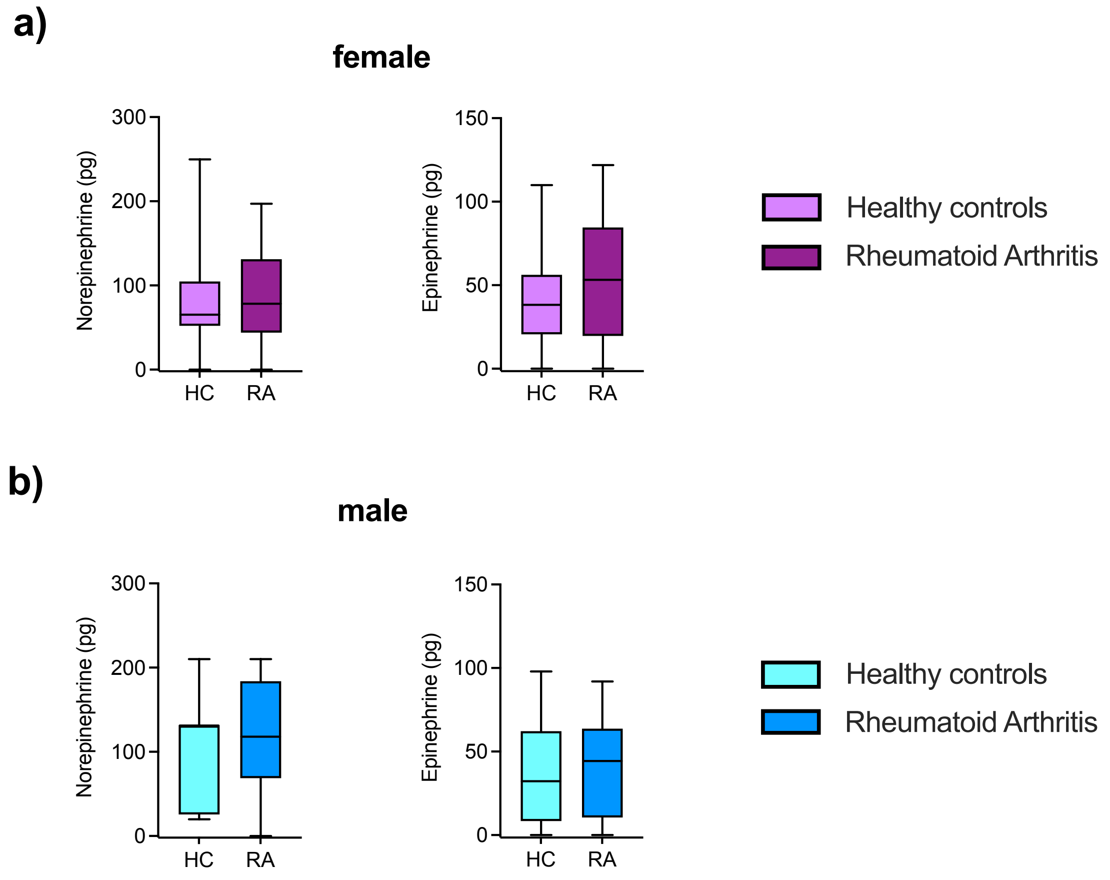
a)
female
Norepinephrine (pg)
300
200
100
0
HC
RA
Epinephrine (pg)
150
100
50
0
HC
RA
Healthy controls
Rheumatoid Arthritis
b)
male
Norepinephrine (pg)
300
200
100
0
HC
RA
Epinephrine (pg)
150
100
50
0
HC
RA
Healthy controls
Rheumatoid Arthritis

**a)**

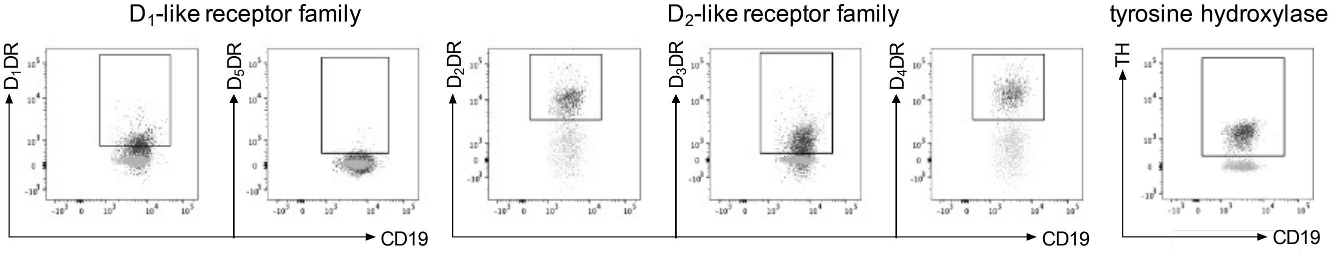
D1-like receptor family
D2-like receptor family
tyrosine hydroxylase
D1DR
D5DR
D2DR
D3DR
D4DR
TH
CD19

**b)**

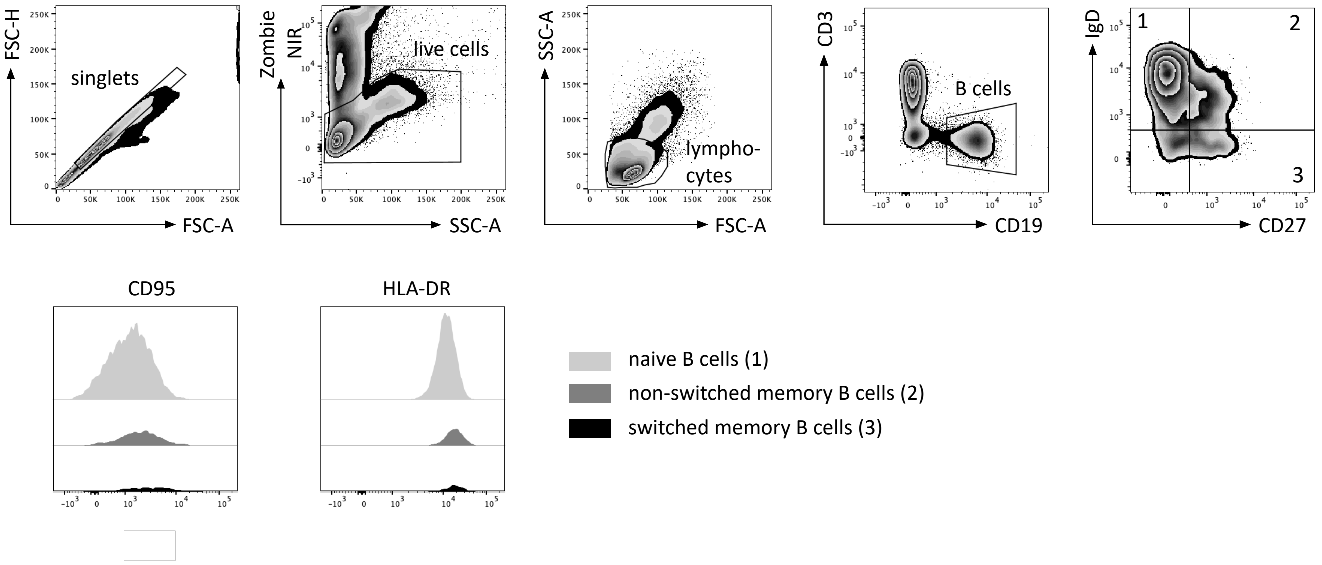
FSC-H
singlets
FSC-A
Zombie NIR
live cells
SSC-A
lympho-
cytes
CD3
B cells
CD19
IgD
1
2
3
CD27
CD95
HLA-DR
naive B cells (1)
non-switched memory B cells (2)
switched memory B cells (3)

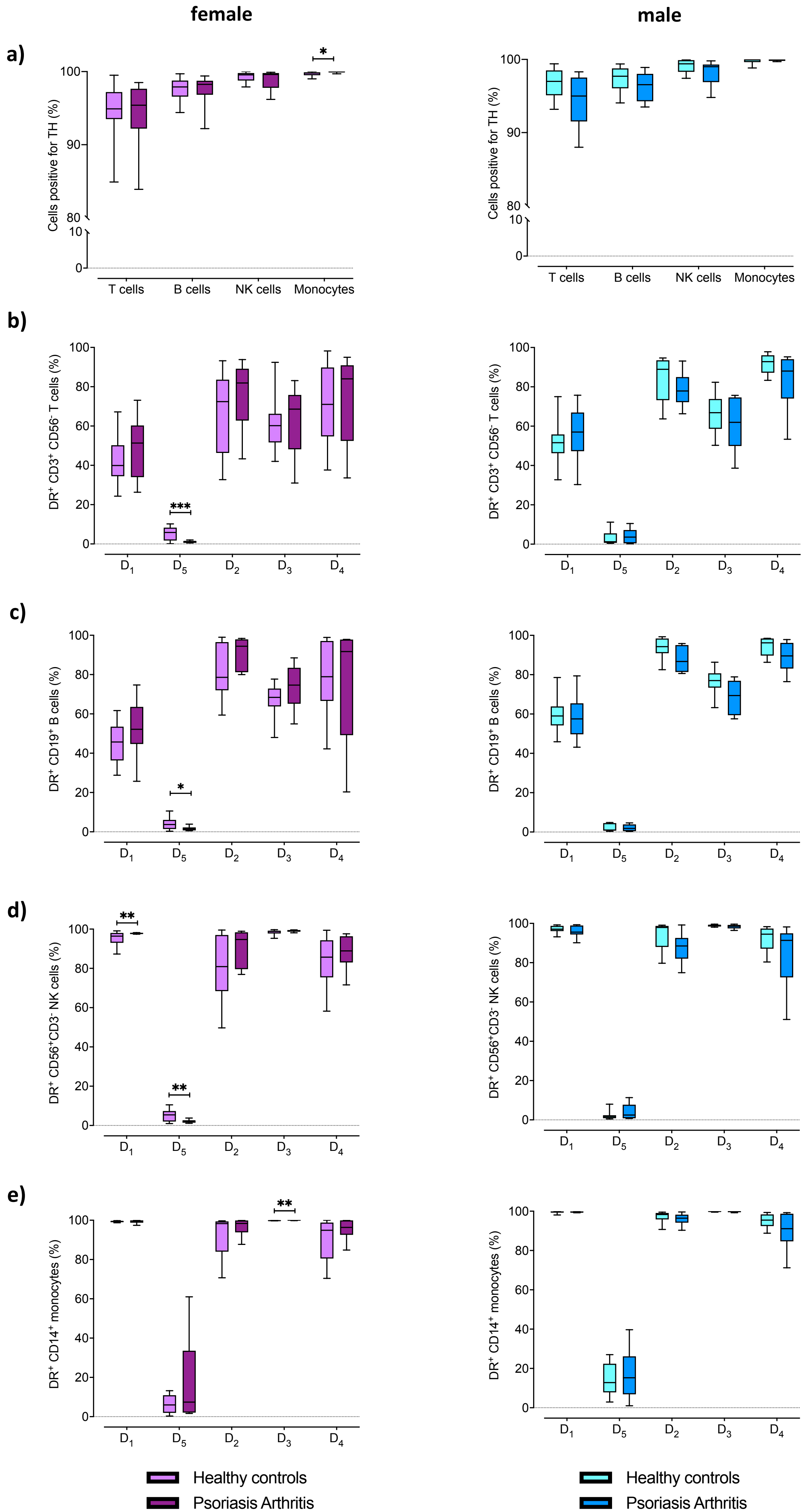
female
male
a)
Cells positive for TH (%)
T cells
B cells
NK cells
Monocytes
b)
$DR^+ CD3^+ CD56^- T$ cells (%)
$D_1$
$D_5$
$D_2$
$D_3$
$D_4$
c)
$DR^+ CD19^+$ B cells (%)
d)
$DR^+ CD56^+ CD3^-$ NK cells (%)
e)
$DR^+ CD14^+$ monocytes (%)
Healthy controls
Psoriasis Arthritis

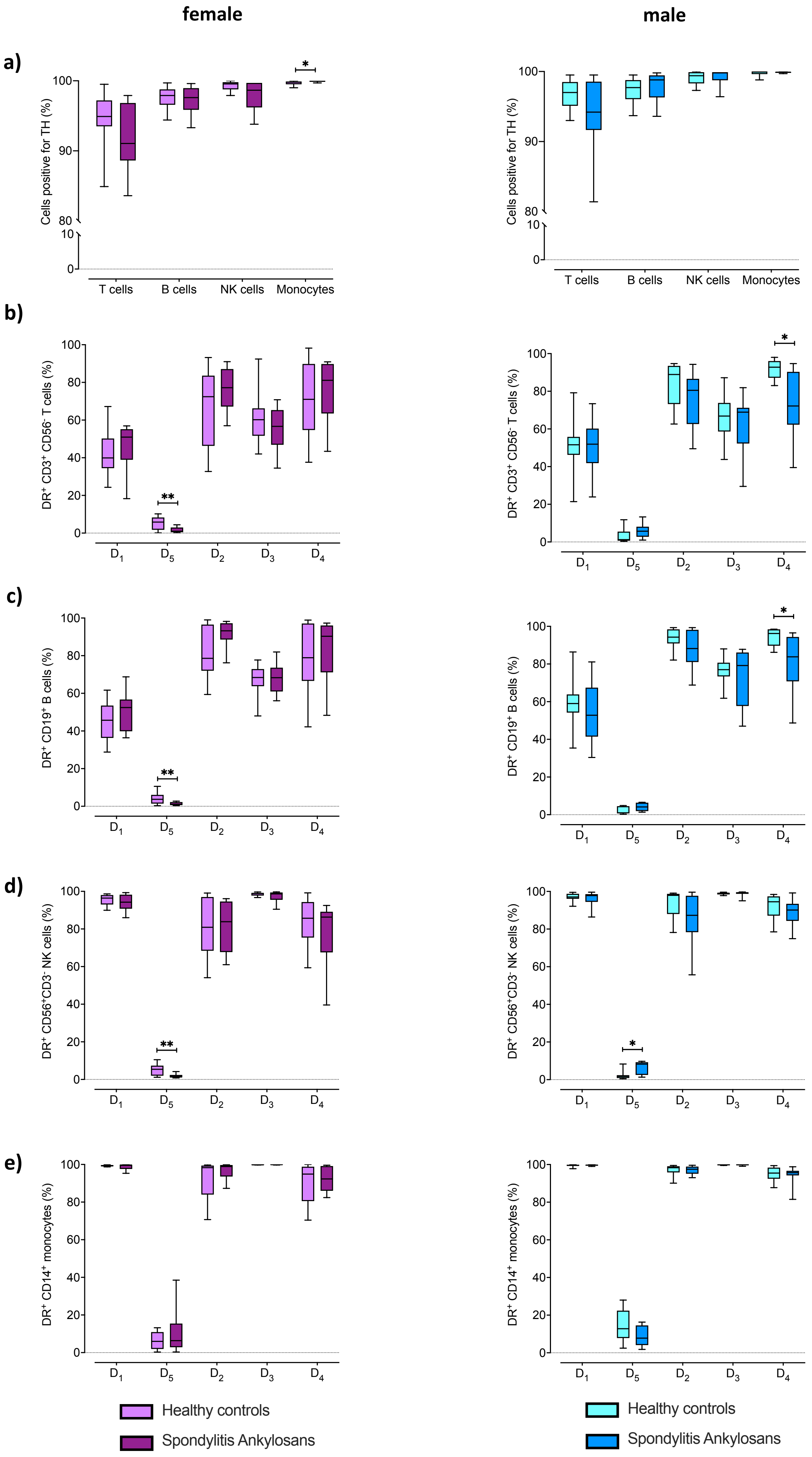
female
male
a)
Cells positive for TH (%)
T cells
B cells
NK cells
Monocytes
b)
DR+ CD3+ CD56- T cells (%)
c)
DR+ CD19+ B cells (%)
d)
DR+ CD56+CD3- NK cells (%)
e)
DR+ CD14+ monocytes (%)
D1
D5
D2
D3
D4
Healthy controls
Spondylitis Ankylosans

**a)**

**female**

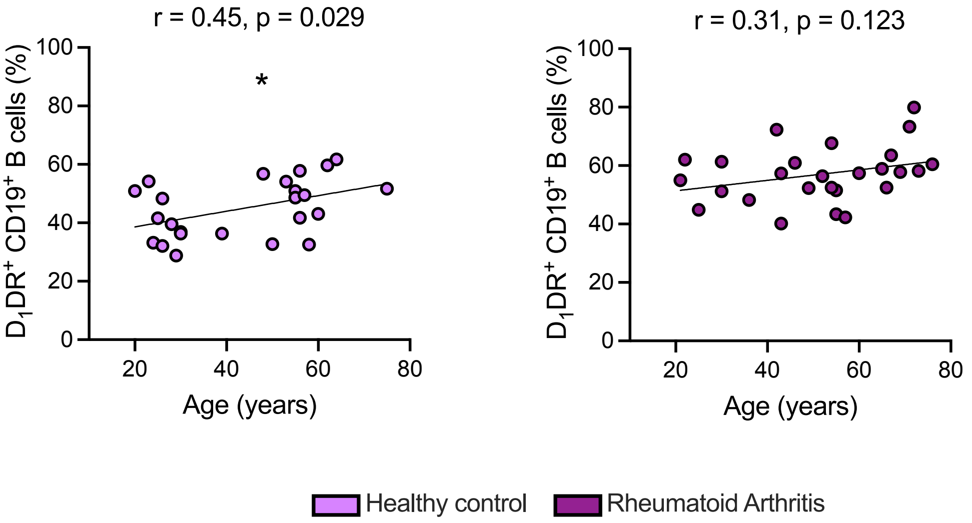


**b)**

**male**

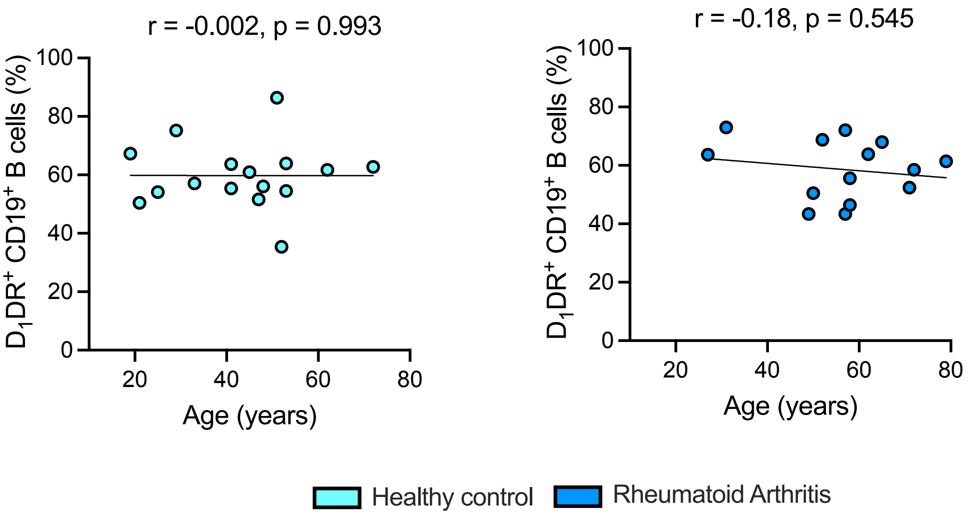

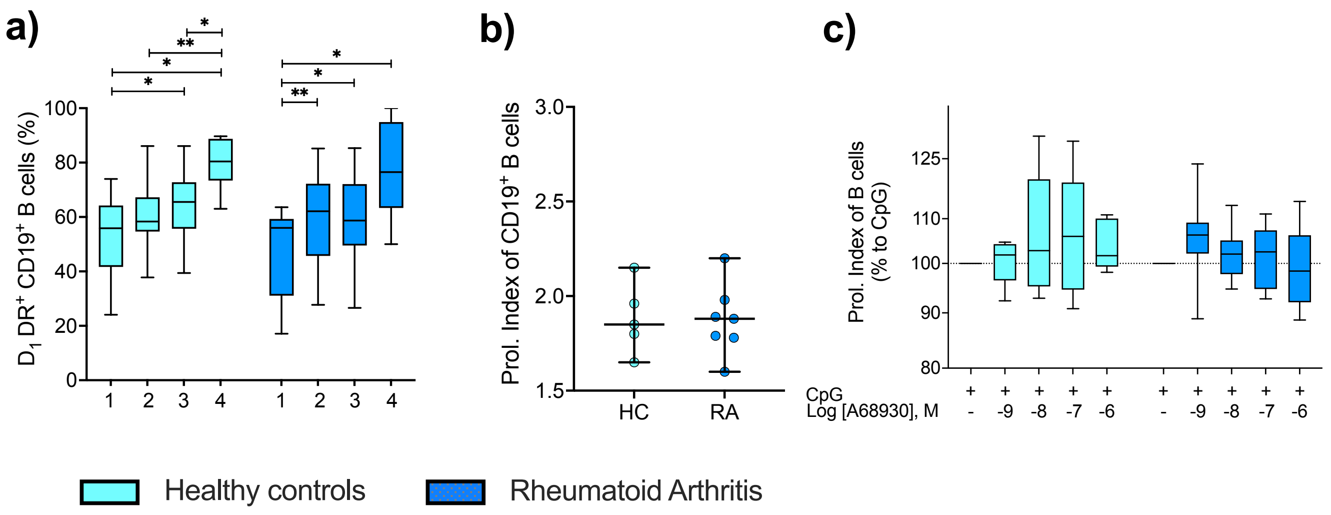
a)
D1 DR+ CD19+ B cells (%)
0
20
40
60
80
100
1 2 3 4 1 2 3 4
*
**
*
*
*
*
**
b)
Prol. Index of CD19+ B cells
1.5
2.0
2.5
3.0
HC
RA
c)
Prol. Index of B cells (% to CpG)
80
90
100
110
125
CpG + + + + + + + + + +
Log [A68930], M - -9 -8 -7 -6 - -9 -8 -7 -6
Healthy controls
Rheumatoid Arthritis

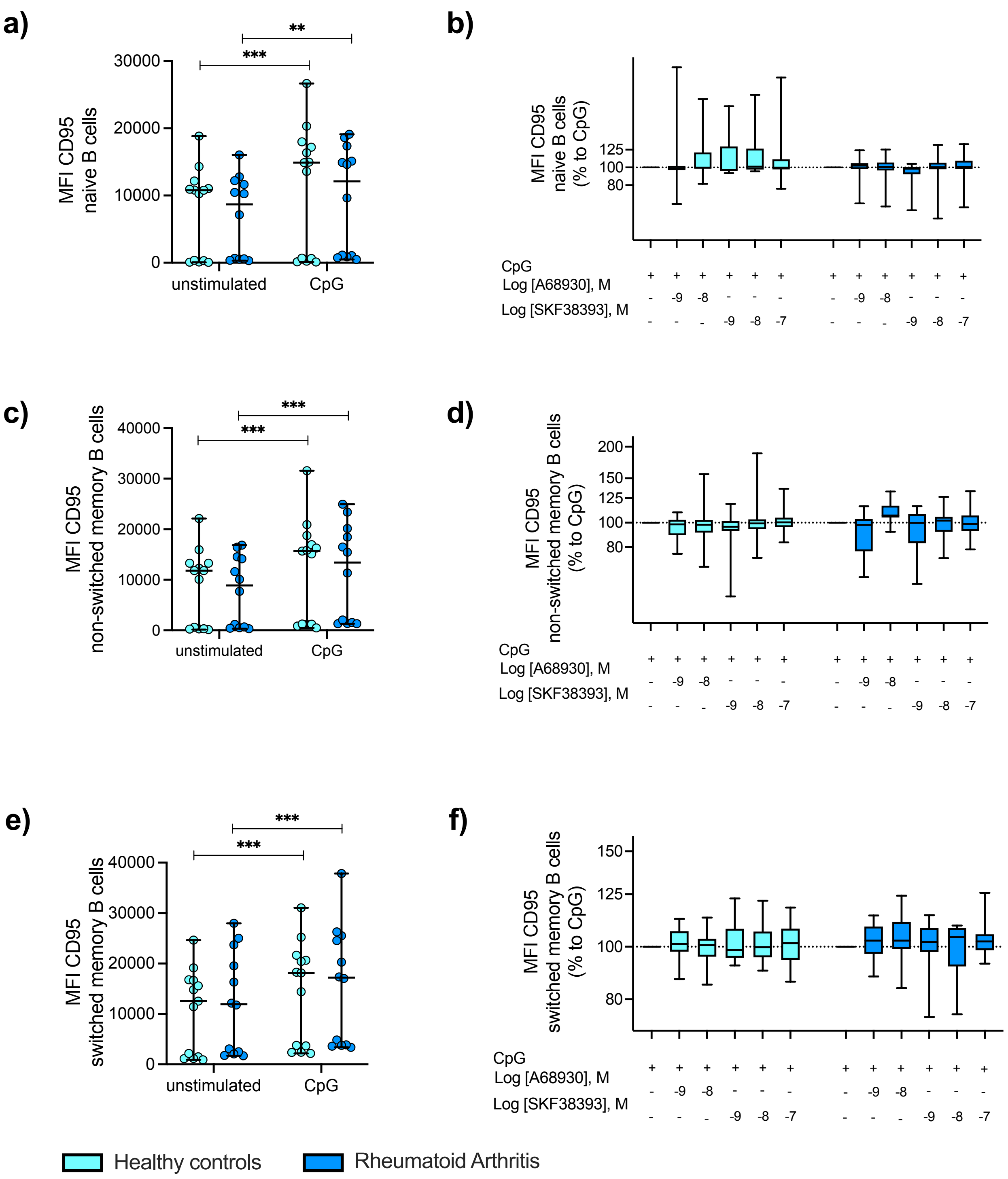
a)
MFI CD95
naive B cells
30000
20000
10000
0
***
**
unstimulated
CpG
b)
MFI CD95
naive B cells
(% to CpG)
125
100
80
CpG
Log [A68930], M
Log [SKF38393], M
c)
MFI CD95
non-switched memory B cells
40000
30000
20000
10000
0
***
***
unstimulated
CpG
d)
MFI CD95
non-switched memory B cells
(% to CpG)
200
150
125
100
80
CpG
Log [A68930], M
Log [SKF38393], M
e)
MFI CD95
switched memory B cells
40000
30000
20000
10000
0
***
***
unstimulated
CpG
f)
MFI CD95
switched memory B cells
(% to CpG)
150
125
100
80
CpG
Log [A68930], M
Log [SKF38393], M
Healthy controls
Rheumatoid Arthritis

Suppl. Figure 8

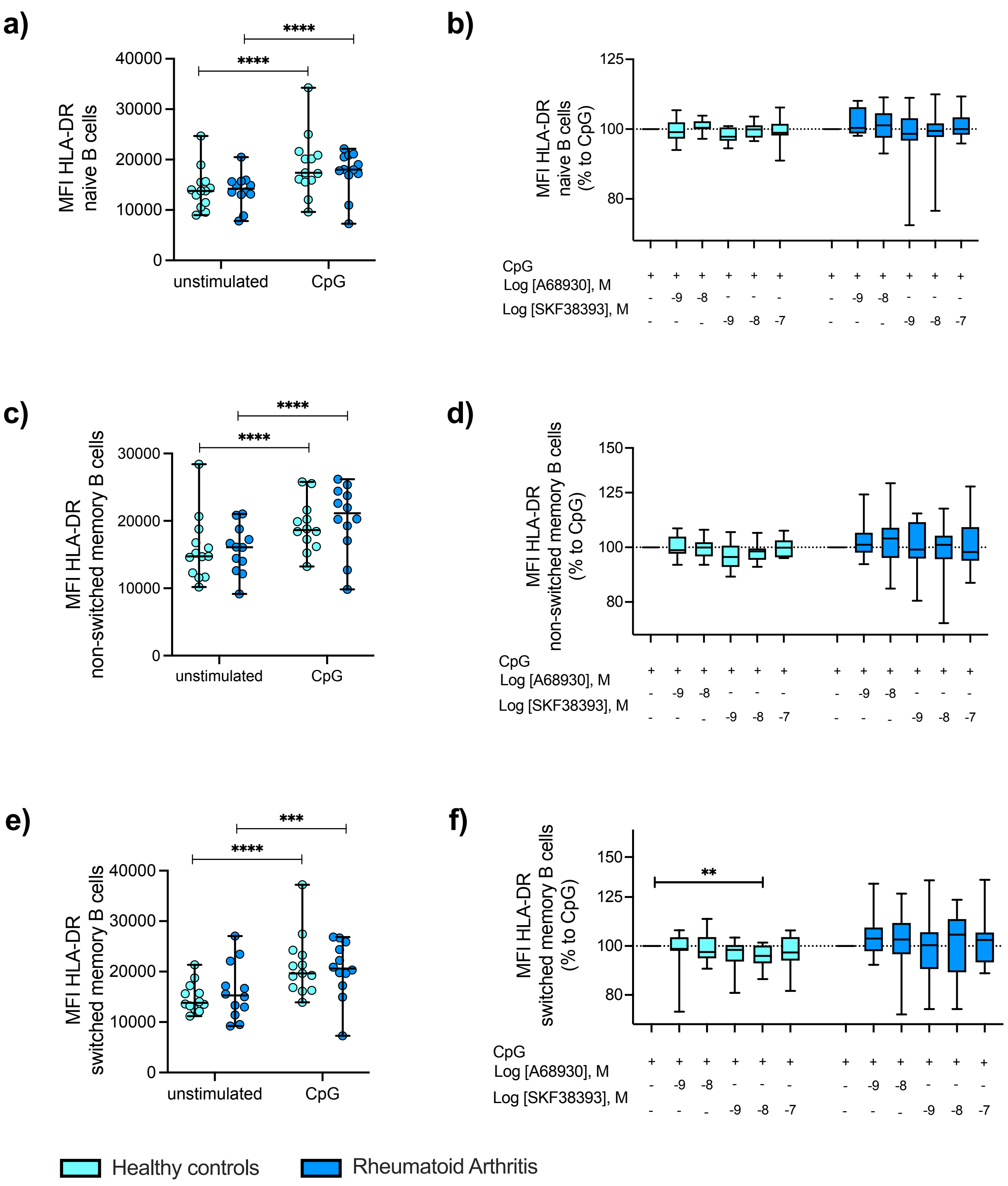

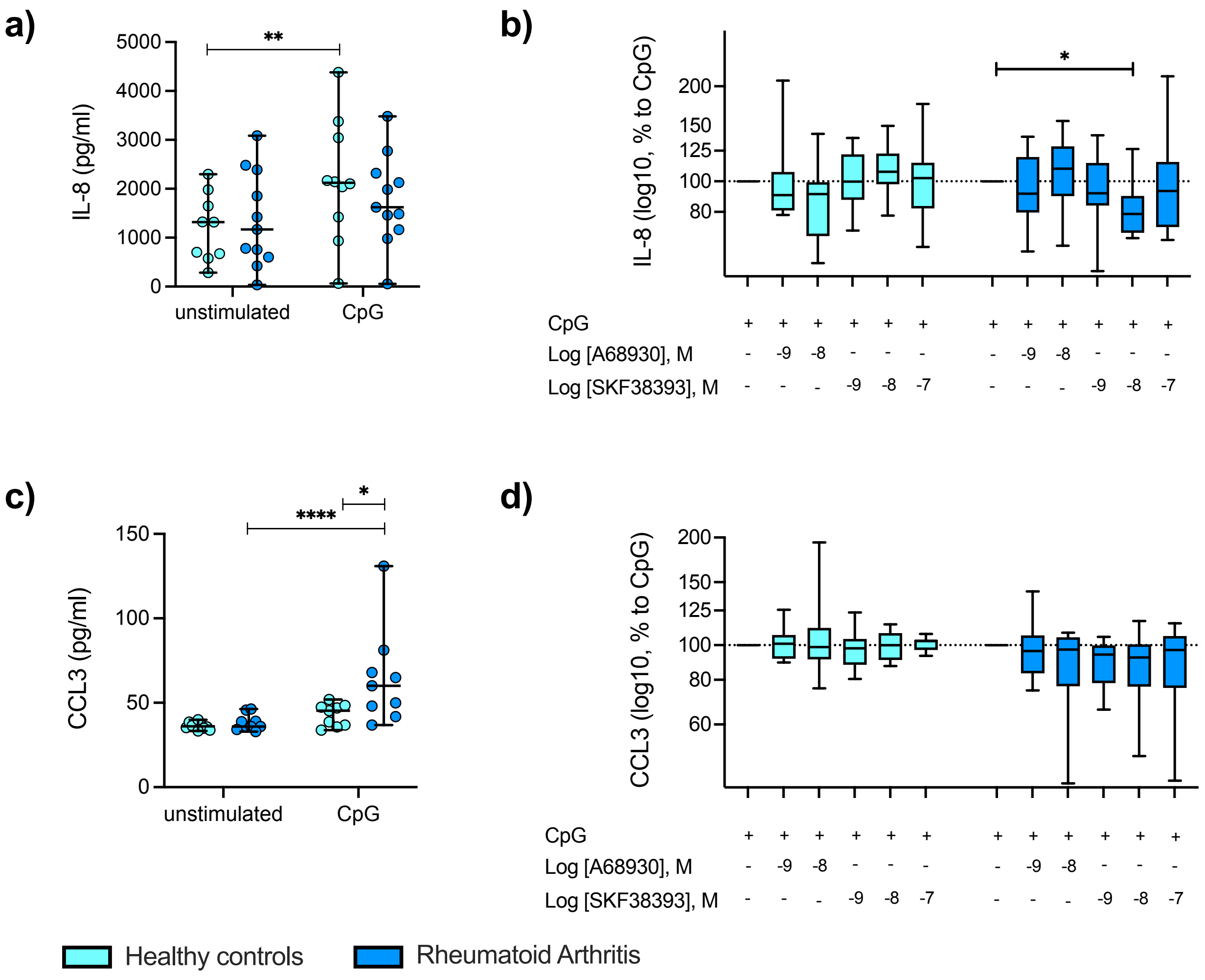
a)
IL-8 (pg/ml)
5000
4000
3000
2000
1000
0
**
unstimulated
CpG
b)
IL-8 (log10, % to CpG)
200
150
125
100
80
*
CpG + + + + + + + + + + + +
Log [A68930], M - -9 -8 - - - - -9 -8 - - -
Log [SKF38393], M - - - -9 -8 -7 - - - -9 -8 -7
c)
CCL3 (pg/ml)
150
100
50
0
****
*
unstimulated
CpG
d)
CCL3 (log10, % to CpG)
200
150
125
100
80
60
CpG + + + + + + + + + + + +
Log [A68930], M - -9 -8 - - - - -9 -8 - - -
Log [SKF38393], M - - - -9 -8 -7 - - - -9 -8 -7
Healthy controls
Rheumatoid Arthritis
